# Recent advances of metal-organic frameworks (MOFs) for drug delivery, cancer imaging and theranostics

**DOI:** 10.7150/thno.128435

**Published:** 2026-02-26

**Authors:** Honglian Yu, Gan Lin, Peng Mi

**Affiliations:** 1 Department of Radiology and State Key Laboratory of Biotherapy, West China Hospital, and College of Polymer Science and Engineering, Sichuan University, No.17 South Renmin Road, Chengdu, Sichuan 610041, China.; 2 Department of Biomedical Engineering, NanoSTAR Institute, University of Virginia School of Medicine, Charlottesville, VA 22908, USA.

**Keywords:** metal-organic frameworks, nanocarriers, drug delivery, theranostics, molecular imaging.

## Abstract

Metal-organic frameworks (MOFs) are a unique class of porous materials constructed from metal-containing nodes, known as secondary building units (SBUs) and organic ligands. Their highly tunable structures enable the encapsulation of a broad range of therapeutic agents, spanning small-molecule chemotherapeutics to biomacromolecules such as proteins, DNA, and RNA. By rational selection of metal ions and organic linkers, diverse functionalities, including molecular imaging and phototherapeutic capabilities, can be included into MOFs, rendering them promising nanoscale platforms of nanomedicines. In this review, we summarize recent advances of MOFs for drug delivery, cancer imaging and theranostics. We discuss the progress in regulating the morphology and functions of MOFs through diverse synthetic strategies and surface modification approaches. We further systematically analyzed and discussed MOFs in the applications of drug delivery, molecular imaging, and cancer theranostics, with recent strategies. Finally, key limitations associated with the clinical translation of MOFs are discussed, along with the corresponding bottlenecks, future challenges, and emerging opportunities.

## Introduction

Cancer remains one of the leading causes of mortality worldwide. The development of effective therapeutic strategies is therefore critically needed 1. However, conventional anticancer drugs often suffer from low bioavailability and significant side effects. To enable precise cancer therapy with enhanced therapeutic efficacy, a wide range of nanocarriers have been developed over recent decades, including organic systems such as dendrimers, micelles, and liposomes, as well as inorganic platforms such as magnetic nanoparticles, quantum dots, and metal nanoparticles 2,3. Nanocarriers can be functionalized through interdisciplinary approaches for cancer molecular imaging, therapy, and theranostics 4,5. Consequently, their biomedical applications have expanded rapidly, encompassing platforms such as liposomes, polymeric nanoparticles, MOFs, and covalent organic frameworks (COFs).

Recently, MOFs offer distinct advantages, including exceptionally high drug-loading capacity arising from their large surface area, as well as versatile functionalization via ligand modification or metal-node substitution. These features allow MOFs to function as integrated theranostic platforms. Since the seminal work by Hoskins and Robson elucidating the structures and anion-exchange properties of porous coordination polymers, this class of materials has rapidly evolved into a major research field 6,7. The term of MOF was introduced by Yaghi and co-workers in 1995 8, and subsequently established the standardized terminology by the International Union of Pure and Applied Chemistry (IUPAC) in 2013. Over the past decades, MOFs have been widely explored for applications in catalysis 9, gas storage 10, energy-related technologies 11, and drug delivery 12.

MOFs have demonstrated considerable promise across a broad range of biomedical applications, including molecular imaging, drug delivery, and theranostics. This potential arises from their intrinsic physicochemical properties, such as tunable porosity, customizable architectures, high drug-loading capacity, partial biodegradability, and, in certain systems, acceptable biocompatibility. Owing to their highly porous architecture and large specific surface area, MOFs enable efficient encapsulation and controlled release of diverse therapeutic agents 13. To data, MOFs have demonstrated efficient loading for a spectrum of cargos, including small-molecule anticancer drugs, nucleic acids, *e.g.*, short interfering RNA (siRNA) and DNA, contrast agents 14 for various applications including gene therapy 15, chemotherapy 16, immunotherapy 17,18, *etc*.

Beyond drug delivery, MOFs and their derivatives have been explored as molecular imaging probes or as carriers for imaging agents across multiple modalities, including fluorescence imaging (FL) 19, magnetic resonance imaging (MRI) 20, computed tomography (CT) imaging 21, positron emission tomography (PET) imaging 22, and photoacoustic imaging (PAI) 23. Such versatility is achieved through rational selection of metal nodes and organic linkers or through the incorporation of dedicated imaging moieties. The integration of therapeutic agents within MOF-based carriers can further enhance cellular uptake, gene silencing, and overall therapeutic performance through rational structural and compositional design 24.

More recently, MOFs constructed by photosensitizer (PS) or radiosensitizer ligands have been reported to significantly enhance the efficacy of photodynamic therapy (PDT) and radiotherapy (RT) 25. In addition, MOFs can act as versatile carriers for anticancer agents, enabling combinatorial and synergistic treatment strategies. Within the tumor microenvironment (TME), MOFs, typically with particle sizes in the range of 20-200 nm and favorable aqueous dispersibility, can preferentially accumulate at tumor sites via the enhanced permeability and retention (EPR) effect 26, thereby improving tumor targeting while mitigating off-target toxicity to healthy tissues 27. Consequently, the multifunctional design of MOFs offers distinct advantages in the TME, facilitating precise delivery and controlled release of chemotherapeutics or imaging agents, ultimately enhancing both therapeutic efficacy and diagnostic accuracy.

Despite their promise, MOFs exhibit limitations compared with other nanocarriers, due to potential toxicity of heavy metal ions. Consequently, the choice of MOF nanocarrier must be guided by application-specific requirements—including drug characteristics, target tissues, administration routes—as well as safety and manufacturability considerations.

**Figure 1:**
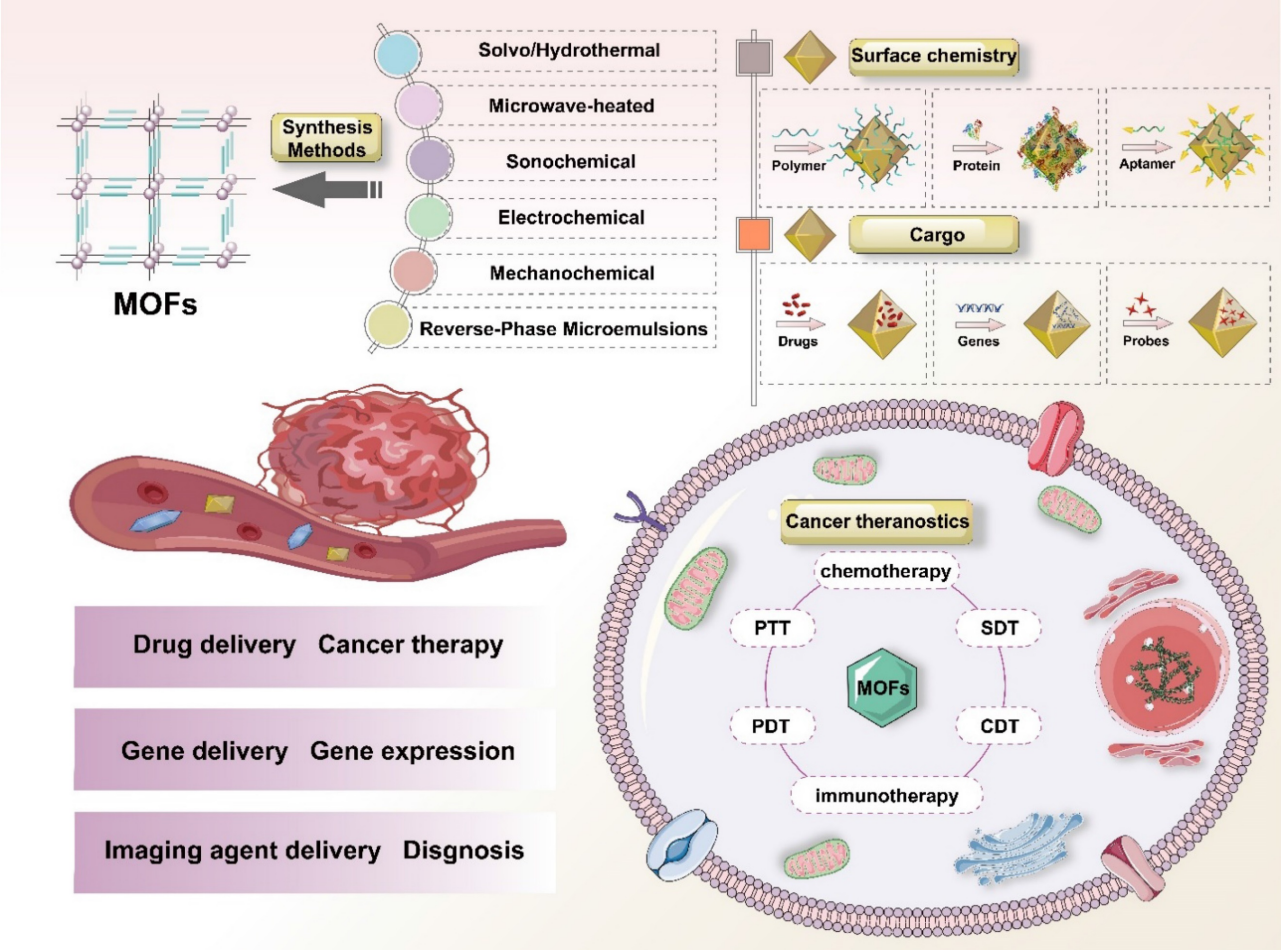
Schematic illustration of MOFs and their biomedical applications for drug delivery, cancer molecular imaging, and theranostics.

Herein, we provide a comprehensive overview of recent advances in synthetic methodologies and biomedical applications of MOFs for cancer treatment, with particular emphasis on drug delivery mechanisms, molecular imaging capabilities, and theranostic potentials (Figure 1). Additionally, we highlight key challenges and future perspectives in this rapidly evolving field, with the aim of guiding further research and innovation in MOF-based cancer nanomedicine.

## Methods for preparing MOFs

**Figure 2:**
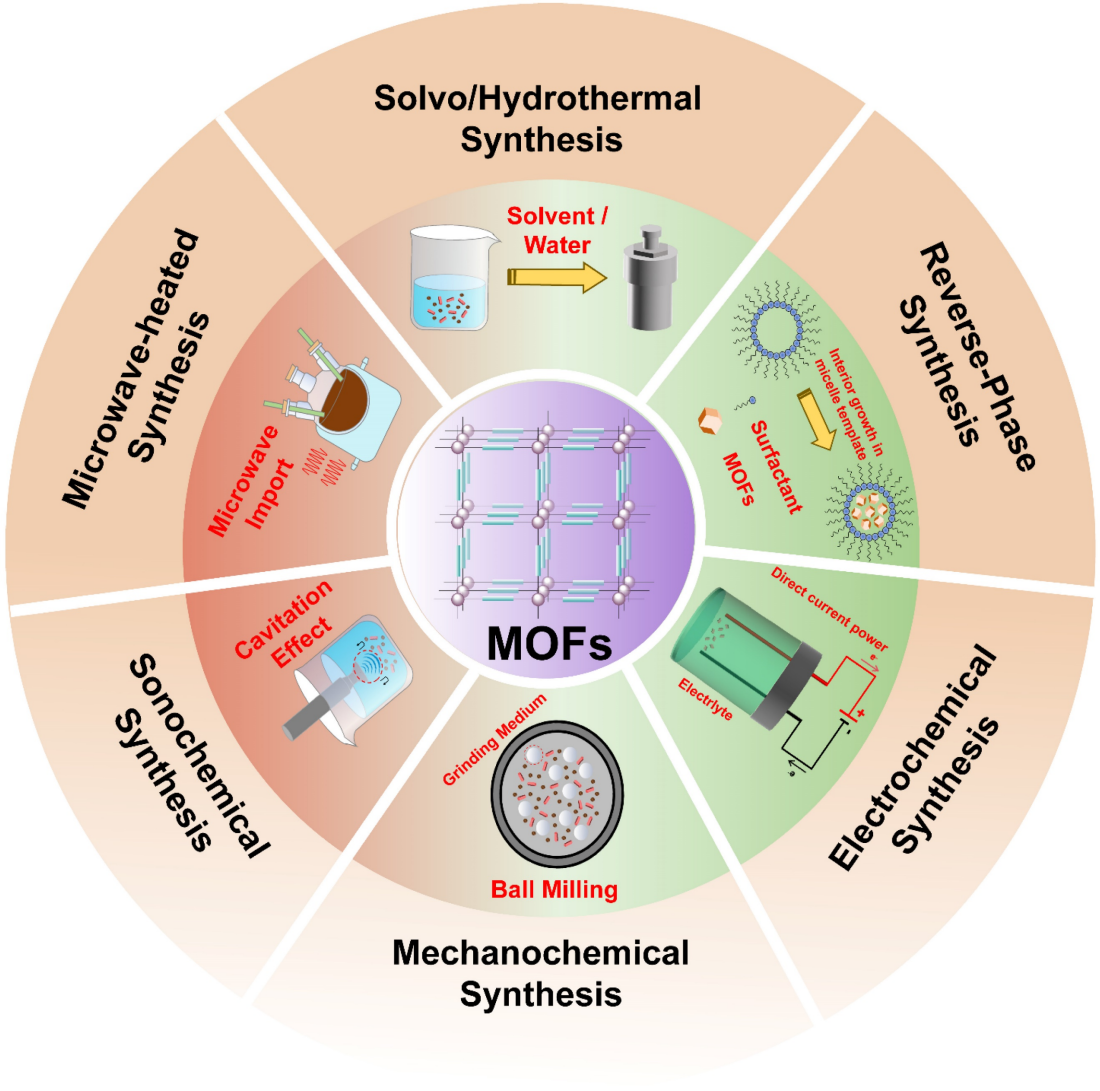
** Schematic summary of classical methods for the synthesis of MOFs:** Solvo/Hydrothermal synthesis, Microwave-heated synthesis, Electrochemical synthesis, Sonochemical synthesis, Mechanochemical synthesis, and Reverse-phase synthesis.

The publication of a seminal 1989 study by Robson describing the self-assembly of MOFs through the coordination of metal ions or clusters with organic ligands stimulated broad interest in these materials within the scientific community 7. In 1994, the first three-dimensional metal-porphyrin coordination polymer was synthesized using palladium-based tetrapyridine porphyrin ligands coordinated with Cd^2+^ ions 28. In 1999, Yaghi and co-workers reported MOF-5, which was the first stable and porous MOF, and thereby established MOFs as an independent field of materials research 29. Subsequently, Gérard Férey pioneered the development of large-pore and highly stable MIL frameworks, Kitagawa advanced the study of flexible and dynamic MOFs, and Joe established reticular chemistry as a unifying framework for predicting and classifying MOF structures 30,31. In recent years, several representative MOF families, including MILs, ZIFs, HKUSTs, UiOs, *etc*. (Table 1), have been extensively investigated for biomedical applications. These MOFs are commonly synthesized via solvo/hydrothermal, microwave-heated, sonochemical, electrochemical, mechanochemical and reverse-phase microemulsions syntheses (Figure 2).

### Classical method for synthesizing MOFs

Solvothermal/hydrothermal synthesis is one of the most widely used approaches for MOF fabrication, involving reactions between metal salts and ligands in sealed vessels under elevated temperature and pressure. Reaction parameters, including temperature, pressure, time, pH, precursor concentration, and filling degree, govern crystal size, yield, and morphology. Higher temperatures and pressures accelerate crystallization, while extended reaction times promote crystal growth from the nano- to microscale 45. The introduction of modulators (*e.g.*, acetic, benzoic, or lauric acid) competes with organic ligands during nucleation, enabling precise control over particle size, morphology, and crystallization kinetics. pH optimization is highly system-dependent: acidic conditions weakened diffraction intensity, whereas alkaline environments often led to the formation of phase impurities 46. Recent optimization strategies have further incorporated controlled precursor hydrolysis and the use of polymer or surfactant additives to improve crystallinity and morphology regulation. The MOFs synthesized by solvothermal/hydrothermal approach usually exhibit good crystallinity and stable structure, with tunable pore sizes that enable effective drug loading and delivery. However, the high energy demand of this method limits its large-scale production, and post-synthetic treatments are usually required to obtain nanostructures.

Microwave-assisted synthesis employs electro-magnetic radiation (300 MHz-30 GHz) to directly couple with molecular dipoles, enabling rapid and heating that promotes MOF nucleation and crystallization in sealed high-pressure reactors. This volumetric heating mechanism markedly shortened reaction times and reduced particle sizes compared with conventional solvothermal methods, owing to uniform energy distribution and minimized local overheating 47. For example, MIL-101(Cr) with a uniform particle size of approximately 20 nm can be synthesized within 5 min at 210 °C and 600 W without hydrofluoric acid, whereas traditional methods typically require about 8 h and with HF to yield heterogeneous microscale particles 48. This approach enables the efficient production of small and monodisperse nanoparticles with readily tunable synthesis parameters, rendering it well suited for intravenous administration. However, the presence of thermal gradients under certain conditions may induce structural defects, potentially compromising framework integrity and drug-loading efficiency.

Sonochemical synthesis represents an efficient unconventional method for MOF fabrication. Under ultrasonic irradiation (20 kHz to 10 MHz), acoustic cavitation generates localized extreme conditions (> 5000 K, ~1000 atm) with rapid heating/cooling rates (> 10^10^K s^-1^), which promote homogeneous nucleation and accelerated crystallization 49. Compared with conventional thermal heating, ultrasound delivers energy more uniformly throughout the reaction medium, thereby significantly enhancing MOF crystallization. The first MOF synthesized via the sonochemical route was Zn_3_(BTC)_2_ 50, which was obtained within 5 min with an average particle size of ~90 nm and a high yield of 75.3%. In contrast, the conventional hydrothermal method requires 24 h at 140 °C to produce the same compound 51. Although this method enables relatively rapid synthesis of small-sized MOFs, it often suffers from reduced crystallinity, high sensitivity to ultrasonic parameters, and potential formation of amorphous or defective structures, which may compromise drug-loading stability.

Electrochemical synthesis encompasses multiple strategies, including cathodic/anodic synthesis, bipolar electrodeposition, potential shift, and electrophoretic deposition. These approaches rely on electrode-electrolyte interfacial reactions under precisely controlled current or potential conditions. Among them, cathodic reduction and anodic oxidation are widely employed due to their mild operation conditions, accurate potential control, and minimal by-product formation 52. Electrochemical synthesis was first applied to fabricate HKUST-1 (Cu_3_(BTC)_2_) films on copper substrates 53. This technique offers several advantages, including short reaction times, benign reaction conditions, high product purity (owing to the absence of metal salts or anionic residues), and tunable morphology, rendering it well suited for continuous synthesis. Using this technique, MOFs such as ZIF-8(Zn), NH_2_-MIL-53, HKUST-1, MIL-53(Al), and MIL-100(Al) have been successfully prepared. Moreover, electrochemical synthesis is typically conducted in a solvent-free or aqueous system, providing favorable biocompatibility. Precise control of electrochemical parameters enables the formation of MOFs with stable pore architectures and high drug-loading capacities. However, the resulting MOFs are predominantly as thin films, which limits their applicability to specific drug delivery formats, such as implantable device coatings or wound dressings.

Mechanochemical synthesis employs mechanical forces, such as shear, friction, or compression to induce reactions between metal salts and organic ligands, leading to MOF formation without the need for bulk solvents. This solvent-free or solvent-minimized approach enables scalable powder production with reduced environmental impact and lower cost compared with conventional solvent-based methods. Typically, synthesis is conducted in in grinding jars containing balls, metal ions precursors, organic ligands, and additives. The first mechanochemically synthesized MOF was obtained by manually grinding metal oxides with imidazole ligands 54. Subsequent methodological advances led to the development of liquid-assisted grinding (LAG) and ion- and liquid-assisted grinding (ILAG). In LAG, small amounts of solvent are introduced to accelerate reaction kinetics and improve crystallinity, whereas ILAG further incorporates inorganic salts to enhance precursor dissolution, reaction homogeneity, and grinding efficiency 55. Overall, mechanochemical synthesis offers a green and scalable route for MOF production, with simplified post-synthetic processing that facilitates drug loading and delivery. However, MOFs produced via this method often exhibit lower porosity than those synthesized by alternative routes, which could limit their drug-loading capacity.

Microemulsions are thermodynamically stable dispersions formed by emulsifying immiscible liquids into dispersed droplets (typically 5-100 nm), resulting in transparent or translucent systems composed of oil, water, surfactants, and cosurfactants. In reverse-phase microemulsion synthesis, surfactants self-assemble into micelles, microemulsions, or liquid-crystals phases that act as organic templates, providing confined nanoscale reactors for MOF formation. Metal ions and ligands react at the droplet interface, yielding MOFs nanoparticles with controlled dimensions. By tuning the surfactants-to-water ratio, the size and morphology of the resulting MOFs could be precisely regulated 56. The approach has been successfully extended to widely applied to MOFs, such as zinc-based frameworks 57. Precise control over the “water-core” size enables the synthesis of small, highly monodisperse MOF nanoparticles and allows *in situ* loading of either hydrophobic or hydrophilic drugs according to their physicochemical properties. The confinement effect promotes uniform drug distribution within MOF pores and high encapsulation efficiency. Nevertheless, complete removal of surfactants and solvents, which may induce toxicity, remains a major challenge.

Advances in micro- and nanotechnology have provided more synthetic strategies of MOFs, including microfluidics-assisted and ionthermal methods 58,59. For example, Fe-TCPP MOFs fabricated via droplet-based microfluidics and subsequently functionalized with oxaliplatin prodrugs through ligand exchange exhibited precise size and morphology control, improved batch-to-batch reproducibility, and enhanced encapsulation efficiency. Ionthermal synthesis, which employs ionic liquids as both solvents and structure-directing agents, facilitates the preparation of highly stable MOFs, such as NH_2_-MIL-53-Al, offering high porosity and proton conductivity, while benefiting from intrinsic conductivity and green characteristics of ionic liquids. These techniques have also been extended to the synthesis of spherical porous carbon nanoparticles (SPCNs) and other porous carbon materials derived from MOF templates 60.

In addition, MOF-derived single-atom catalysts (SACs), characterized by atomically dispersed metal active centers, have attracted increasing attention. The low-coordination environments of exposed unsaturated metal atoms maximize catalytic activity; however, suitable substrates are usually required to prevent atom aggregation and maintain the uniform dispersion and long-term stability 61. Among the available strategies, high-temperature pyrolysis remains the most widely used approach, involving thermal decomposition of MOFs under controlled gas atmospheres to generate isolated metal atoms embedded within carbonaceous matrices. For instance, Pd nanoparticles can be transformed into Zn-MOF-based Pd single-atom catalysts by pyrolysis at 900 °C for 3 h under an inert atmosphere using ZIF-8 as a template 62.

Despite the wide range of synthesis routes explored, no single method is universally optimal. Each approach presents distinct advantages and limitations in terms of reaction conditions, particle morphology, size control, uniformity, scalability, and environmental impact. Therefore, the selection of an appropriate synthesis technique depends on the specific requirements of the intended MOF application. As summarized in Table 1, the choice of synthesis method critically influences MOF structure, functionality, and performance. In practice, integrating complementary techniques to offset the limitations of individual approaches has emerged as an effective strategy for producing high-quality MOF materials with tailored properties.

Although various synthetic methods for MOFs have been developed, the key factors governing MOF synthesis are highly interconnected. These factors include both compositional parameters, such as solvents, reactants, and solution pH, and process parameters, including reaction temperature, pressure, and duration. By rationally modulating these variables, the structures and properties of MOFs could be effectively tailored for biomedical applications. Therefore, careful optimization of relevant parameters and selection of appropriate synthetic methods are essential to meet application-specific requirements.

With the exception of mechanochemical synthesis, most MOF preparation routes begin with dissolving metal precursors and organic ligands in suitable solvents. The influence of the solvent on MOF formation is primarily reflected in reactant solubility, solvent polarity and coordination ability, as well as potential templating effect. These factors need to be considered collectively to identify solvents that facilitate controlled crystal nucleation and growth. Variations in the chemical structures of metal precursors and ligands, together with adjustments in their concentration ratios, can significantly alter the resulting MOF topology. In general, decreasing precursor concentration reduces particle size; however, excessive dilution may lead to particle aggregation and morphological heterogeneity. Because reactant selection varies across synthesis methods, careful control of the precursors is particularly critical for achieving particle sizes suitable for biological applications. For example, mechanochemical synthesis commonly employs metal oxides as precursors. The solution pH strongly influences MOF synthesis by modulating organic ligand solubility, impurity activity, and the degree of ligand deprotonation, thereby indirectly affecting the metal-ligand coordination and crystal growth 63.

Process parameters, including temperature, pressure, and their respective ramping profiles, further influence crystallization kinetics and functional group coordination. Among these available approaches, hydrothermal synthesis offers a distinct advantage by enabling reactions under elevated temperature and pressure, thereby overcoming the poor solubility of certain reactants under ambient conditions and promoting crystallization. Reaction duration also plays a critical role in determining particle size and may lead to aggregation. Accordingly, the choice of synthesis method should be guided by the desired MOF characteristics and reasonable reaction time, with solvothermal methods generally requiring longer synthesis durations.

Ultimately, the biological fate and therapeutic efficacy of MOFs in drug delivery applications are fundamentally dictated by their intrinsic chemical composition, including the nature of metal nodes, organic linkers, pore architecture, and structural defects. These features collectively determine their stability, biocompatibility, targeting ability, drug loading and release kinetics, and clearance behavior *in vivo*. The selection of metal nodes is essential: endogenous or therapeutic ions relevant ions (*e.g.*, Zn^2+^ and Fe^3+^) offer improved biosafety and stimuli-responsive degradation, whereas more inert metals (*e.g.*, Zr^4+^), despite their high structural stability, raise concerns regarding long-term accumulation. Organic linkers further modulate surface chemistry, hydrophilicity, and functionality potential. Pore geometry, including size, shape, and surface chemistry, directly governs drug loading capacity and release behavior. Moreover, the deliberate introduction of structural defects can enhance drug loading, facilitate diffusion, and fine-tune degradation kinetics while preserving overall framework integrity. Collectively, the coordinated interplay of MOF chemical components dictates critical biological outcomes: including colloidal stability in circulation, immune evasion, cellular uptake, endosomal escape, stimuli-triggered drug release, and biodegradable clearance.

### AI-assisted prediction of MOF synthesis

Artificial intelligence (AI) has advanced rapidly in recent years, driven by the proliferation of big data and increasing computational power. With continuously improvement, AI has emerged as a powerful tool for addressing complex challenges across multiple aspects of materials science. Accordingly, the design and synthesis of MOFs are increasingly positioned to benefit from AI-assisted approaches 64,65.

AI-enabled MOF development begins with the construction of high-quality databases. Several well-established MOF databases, such as CoRE MOF, SynMOF, and the Cambridge Structural Database (CSD), are now available and can serve as training and validation datasets for machine learning models. These datasets support model development across four major learning paradigms: supervised, unsupervised, semi-supervised, and reinforcement learning, depending on the availability of labeled data. By integrating traditional computational methods with machine learning algorithms, researchers can rapidly screen large numbers of candidate materials, thereby prioritizing the most promising systems for experimental validation and significantly reducing trial-and-error efforts 66,67. The structures and functions of MOFs can be systematically optimized through modulation of metal nodes, organic linkers, and reaction conditions. AI offers a powerful means to accelerate this optimization by identifying structure-property-performance relationships. For instance, AI-guided screening of microwave-assisted synthesis parameters has been shown to enhance MOF crystallinity, leading to improved material performance 68.

Looking forward, MOF design and synthesis are expected to become increasingly dynamic and impactful through deeper integration with AI and computational technologies. AI-driven strategies are anticipated to accelerate the development of stimuli-responsive MOFs for drug delivery, while also enabling more sustainable and environmentally friendly synthesis pathways. Such advances may yield MOF systems with improved *in vivo* safety, higher drug loading capacity, and enhanced targeting responsiveness, ultimately facilitating their clinical translation.

## Surface modification of MOFs for biomedical applications

The surface chemistry of MOFs plays a critical role in determining their application, as it governs key processes including targeting and uptake. Furthermore, numerous MOFs are susceptible to hydrolysis, framework dissociation, or ligand exchange under physiological conditions. Moreover, unmodified MOF surfaces are prone to nonspecific adsorption of plasma proteins, leading to the formation of a “protein corona”. This corona accelerates clearance and compromises targeting ability. Hence, surface functionalization has been widely employed to augment structural stability in physiological conditions and to enable the conjugation of therapeutic agents for site-specific delivery. In biomedical applications, surface functionalization strategies generally adhere to three fundamental principles: establishing stable interactions between hydrophilic components and the MOF surface, minimizing nonspecific protein adsorption, and enabling controlled and robust attachment for biological functional moieties 69.

### Physical interaction

The first strategy involves physical adsorption of biomaterials, such as polymers or macromolecules on the MOF surface. These interactions are often mediated by electrostatic forces and can enhance MOF properties including structural stability. For instance, electrostatic assembly between negatively charged glucose oxidase and positively charged PCN-222-Fe markedly improves the stability and reusability, thereby yields a new class of chemical and biological catalysts that can be used for biomedical applications 70.

Beyond simple adsorption, additional physical surface modifications include entrapment, self-assembly, and layer-by-layer (LbL) deposition. Entrapment typically involves dissolving target molecules in a solvent together with the host material, followed by controlled expansion and subsequent non-solvent-induced shrinkage of the matrix, thereby physically trapping the molecules. For instance, encapsulating lipase within ZIF-8 via biomineralization preserves enzymatic activity while imparting excellent thermal stability at 50-70 °C 71. Self-assembly refers to the spontaneous organization of discrete building blocks, from individual molecules to structural units, into ordered superstructures. In MOFs, surfactants such as Cetyltrimethylammonium bromide (CTAB) can act as a cationic surfactants and capping agents to produce highly monodisperse, submicron truncated rhombic dodecahedral ZIF-8 colloidal particles, which are suitable for sensing, storage, catalysis, and photonics applications 72. LbL deposition entails the sequential adsorption of molecules or atoms on a substrate, enabling directional, ordered, and controllable MOF surface modification. Although inherently time-consuming, this approach affords precise control over MOF structure and composition while maintaining high crystallinity and structural integrity. For example, *in situ* growth of CuBTC on carboxymethylated cotton via LbL maintains the MOF's crystallinity and microporosity, while simultaneously enhancing water stability and mechanical toughness, thereby broadening its potential applications in self-cleaning textiles and UV shielding 73.

### Chemical modification

Chemical surface modification entails the selective grafting or covalent functionalization of MOFs to tailor their surface chemistry, producing robust coatings suitable for biological applications. Compared with physical interaction, chemical modification offers greater stability and durability, making it particularly advantageous for biomedical use, such as preventing MOF aggregation during circulation and enhancing targeted drug delivery. Common chemical functionalization strategies introduce various surface functional groups (*e.g.*, -OH, -COOH, -NH_2_, -SO_4_^2-^), which regulate hydrophilicity, surface charge, and interactions with protein/cell 74. Surface oxidation introduces oxygen-containing groups, such as carboxyl and carbonyl on MOF surfaces, through the incorporation of peroxide groups. Surface hydrolysis cleaves ester bonds under acidic or alkaline conditions, generating hydroxyl and carboxyl groups. Aminolysis incorporates reactive amino groups, often increasing surface roughness and wettability, thereby modulating interactions with proteins and cells. In addition, plasma treatment generates charged particles by exciting gaseous precursors, which bombard the material surface and induce physicochemical modifications.

MOF surfaces can be functionalized with three main types of biomolecular or synthetic modifiers: polymers, proteins, and aptamer, yielding MOF-polymers, MOF-proteins, and MOF-aptamers systems. Polymers can be further categorized based on their functional roles, including targeting polymers [*e.g.*, folic acid (FA), hyaluronic acid (HA)], polymers with imaging agents (*e.g.*, fluorescent dyes), and polymers-drug conjugates. The incorporation of polymeric coatings substantially enhances MOF stability in biological environments. Notably, polyethylene glycol (PEG) modification usually prolongs blood circulation, increases tumor accumulation via the EPR effect, while markedly reducing protein adsorption and immune clearance. Furthermore, polydopamine-modified MOFs (including HKUST-1-Cu, ZIF-67-Co, ZIF-8-Zn, UiO-66-Zr, Cu-TDPAT, MOF-74-Mg, and MIL-100-Fe), prepared via Michael addition reactions under oxygen (O_2_), exhibit improved structural stability across diverse environments 75. These modifications are crucial for therapeutic applications, such as cancer therapy, by protecting MOFs from premature degradation and maintaining the functional integrity.

MOF-protein conjugates primarily leverage the innate biological functions of proteins to enable active targeting, immune evasion, or catalytic therapy. Proteins are typically attached to MOF surfaces via amide bonds or click chemistry. Alternatively, MOFs could be synthesized on protein templates, or proteins could be incorporated directly as part of the ligand during MOF construction. For instance, covalent conjugation of transferrin to MOF surface enabled specific recognition of the transferrin receptor, which is overexpressed on many tumor cells, thereby facilitating active targeting and significantly enhancing MOF uptake 76. In addition, coating MOFs with intact cell membranes (*e.g.*, via extrusion or sonication) could achieve homologous targeting; for example, cancer cell membranes camouflaged MOFs preferentially accumulate in tumors of the same cellular origin 77.

MOF-aptamer systems utilize single-stranded DNA or RNA aptamers with their high affinity and specificity for selected targets, such as cell surface proteins, small molecules, enabling highly precise cell-level targeting. For example, thiolated AS1411 aptamers have been conjugated to surface-modified, drug-loaded MOFs, allowing selective delivery to cancer cells and promoting their endocytosis 78. In practical applications, combinatorial strategies often yield superior outcomes. For instance, integrating polymer coatings with aptamer functionalization could simultaneously achieve prolonged circulation and precise tumor targeting, representing a promising direction for future MOF surface engineering. Surface modification also plays a pivotal role in enhancing MOF biocompatibility. Phospholipid bilayer-coated Zr-MOFs, formed via Zr-O-P coordination, exhibited enhanced stability, cell uptake, and biocompatibility 79.

Similarly, covalent PEGylation of Zr-MOFs improved their hydrophilicity, aqueous dispersibility, and biological biocompatibility. Surface-modified MOFs demonstrate exceptional performance in targeted drug delivery applications. Among them, the ZIF family, particularly ZIF-8, is widely employed for TME-responsive therapy due to its acid-sensitive degradation. FA-PEG-modified ZIF-8, leverages the overexpression of folate receptors on cancer cells to promotes selective uptake and enables multi-stimuli-responsive drug release 80. Such strategies selectively target cancer cells while minimize off-target toxicity. In addition to drug delivery, MOFs can be functionalized with imaging agents to facilitate real-time monitoring of distribution and therapeutic response. Certain MOFs function directly as contrast agents; for example, Gd-doped polydopamine (PDA) MOFs loaded with PS chlorin e6 (Ce6), enable integrated diagnosis and therapy applications 81. Overall, surface modification strategies endow MOFs with enhanced stability, biocompatibility, targeting precision, and multifunctionality, underscoring their considerable potential in advanced biomedical applications.

## MOFs for drug delivery

Traditional free-form drugs often exhibit unsatisfactory efficacy due to nonspecific biodistribution. Drug delivery systems (DDS) have emerged as a multidimensional strategy designed to enhance drug transport to specific pathological sites (*e.g.*, tumors), thereby improving therapeutic outcomes while minimizing systemic toxicity. The introduction of the first DDS, Spansule®, in 1952, marked the advent of modern controlled-release technologies 86. Over subsequent decades, DDS have successfully addressed many inherent limitations of free drugs, although they have also introduced new challenges related to biocompatibility, stability, and drug-loading efficiency.

As discussed above, DDS research extends beyond the therapeutic agents themselves. Ideal carriers should provide high specificity, robust stability and efficient drug-loading capacity, thereby expanding the clinical applicability of therapeutic agents. MOFs enable drug loading through two principal mechanisms. In the first approach, some drugs themselves can be applied as materials for synthesizing MOFs via strong host-guest or coordination interactions, affording drug protection and enabling controlled release 87. In the second approach, drugs are incorporated into MOF pores through diffusion or adsorption, allowing drug release while maintaining carrier integrity 88. Compared with liposomes and hydrogels, whose drug release depends on carrier degradation or disassembly and often suffers from limited loading efficiency, MOFs offer superior capacity due to their high porosity.

In general, drug release from carriers depends on carrier stability, degradability, and biodistribution. Owing to their large surface area and tunable size, MOFs are particularly well suited for use as drug carriers. Therapeutics can be incorporated into MOFs through diverse mechanisms, including surface adsorption (*e.g.*, electrostatic interactions, coordination bonding, or π-π stacking), pore loading (*e.g.*, PSs, gases, or nanoparticles), and covalent or confinement-based encapsulation of biomacromol-ecules such as proteins and nucleic acids 89. Extensive studies have explored the controlled drug release, biodegradation behavior, and stimulus responsiveness of MOFs, including their sensitivity to both endogenous (*e.g.*, tumor microenvironment) and exogenous (*e.g.*, physical stimuli) triggers 90.

**Figure 3:**
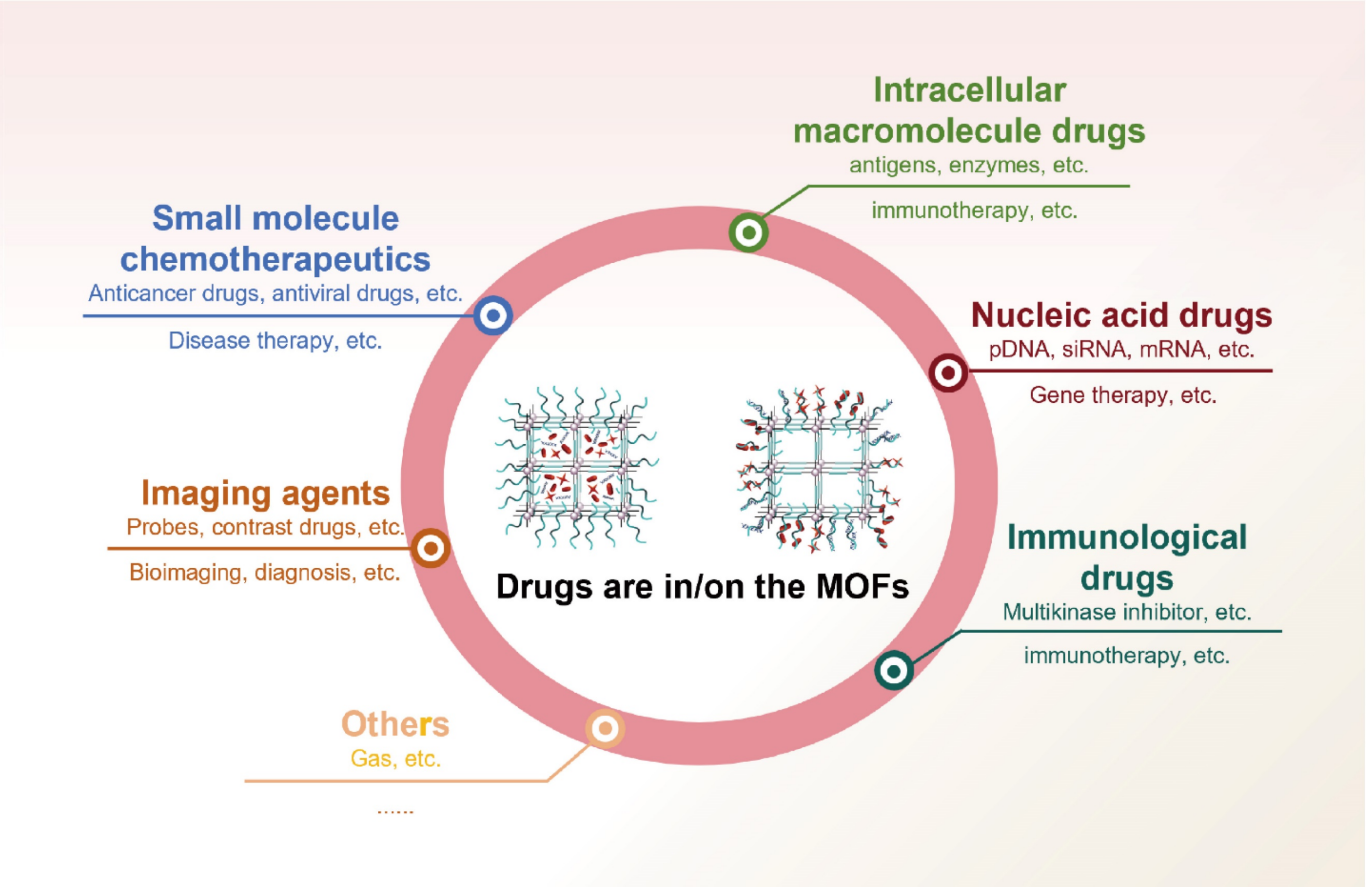
**Applications of MOFs for drug delivery**, including small molecule chemotherapeutics, macromolecules, nucleic acid drugs, immunological drugs, and imaging agents.

This section summarizes recent advances in the application of MOFs as multifunctional carriers for the precise delivery of diverse therapeutic and diagnostic agents, including chemotherapeutics, genes and imaging probes to tumor tissues (Figure 3).

### MOFs for chemotherapeutic compounds delivery

In conventional chemotherapy, many chemotherapeutic agents face significant barriers to clinical application, including poor solubility, chemical instability, low bioavailability, short circulation half-life, nonspecific tissue distribution and systemic toxicity. A fundamental limitation of traditional chemotherapy is the reliance on high drug doses to compensate for inefficient biodistribution, which frequently results in dose-dependent adverse effects. The development of DDS has alleviated several of these challenges by enabling improved targeting and controlled release of chemotherapeutic agents. Among diverse developed DDS platforms, MOFs have emerged as particularly promising carriers owing to their tunable pore architectures, high surface areas, high drug-loading capacities, and controllable multifunctionality. Iron-based MOFs, in particular, have demonstrated favorable biocompatibility and therapeutic efficacy as nanocarriers for the controlled delivery of antitumor and antiviral agents 32. For instance, MIL-100(Fe) has been shown to efficiently encapsulate and deliver bisulfan (25.5%), azidothymidine triphosphate (21.2%), doxorubicin (DOX, 9.1%), and cidofovir (16.1%), enabling effective treatment of both cancer and Acquired Immune Deficiency Syndrome (AIDS). Remarkably, MOFs with diverse architectures have achieved maximum drug-loading efficiencies of up to 81.6 ± 0.6% 91. A summary of representative MOF-based nanocarriers, along with their corresponding cargo types and drug-loading efficiencies (wt%) is presented in Table 2.

In 2008, Horcajada et al. 92 reported two representative MOFs (*i.e.*, MIL-53-Fe, MIL-53-Cr), capable of efficiently loading ibuprofen (IBU). Chemical analyses revealed that both MIL-53(Fe) and MIL-53(Cr) could adsorb approximately 20 wt% of IBU, with sustained release over a three-week period in simulated body fluid (SBF). Owing to its favorable physicochemical properties, MIL-53(Fe) exhibited a higher loading capacity and broader applicability, as further demonstrated with additional cargos such as caffeine (29.2 wt%) 93 and busulfan (18.0 wt%) 94.

**Figure 4:**
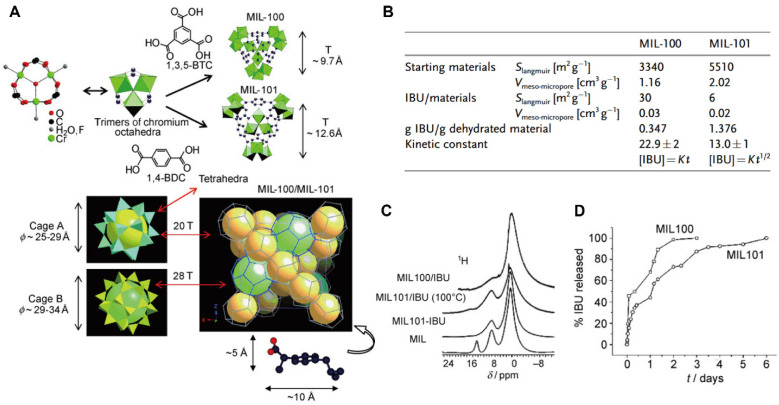
** (A)** 3D schematic of a tetrahedron (T) consisting of a chromium octahedron and 1,4-benzenedicarboxylate moieties or 1,3,5-benzenetricarboxylate groups in MIL-100/MIL-101, respectively; **(B)** Nitrogen adsorption data and the IBU content of MIL-100/MIL-101 investigate; **(C)** ^1^H NMR spectra of MIL-100/IBU and MIL-101/IBU; **(D)** IBU delivery from MIL-100/MIL-101. Adapted with permission from 96, copyright 2006 WILEY-VCH.

Among the first-generation MOFs investigated for drug delivery, the Cr-based MIL-100 and MIL-101 have been extensively studied as classical DDS platforms (Figure 4A) 95. These frameworks exhibited high loading efficiencies for a wide range therapeutics, including azidothymidine triphosphate, cidofovir, DOX, IBU, and caffeine. Notably, IBU, a representative nonsteroidal anti-inflammatory drug, exhibited loading capacities of 0.347 g g^-1^ in MIL-100(Cr) and 1.376 g g^-1^ in MIL-101(Cr) (Figure 4B) 96. The markedly enhanced loading and prolonged release of IBU observed in MIL-101(Cr) were attributed to strong interactions between IBU and Lewis acid metal sites within the framework (Figure 4C, D). Despite these advantages, Cr-based MOFs present notable cytotoxicity, which limits their direct biomedical translation.

**Figure 5:**
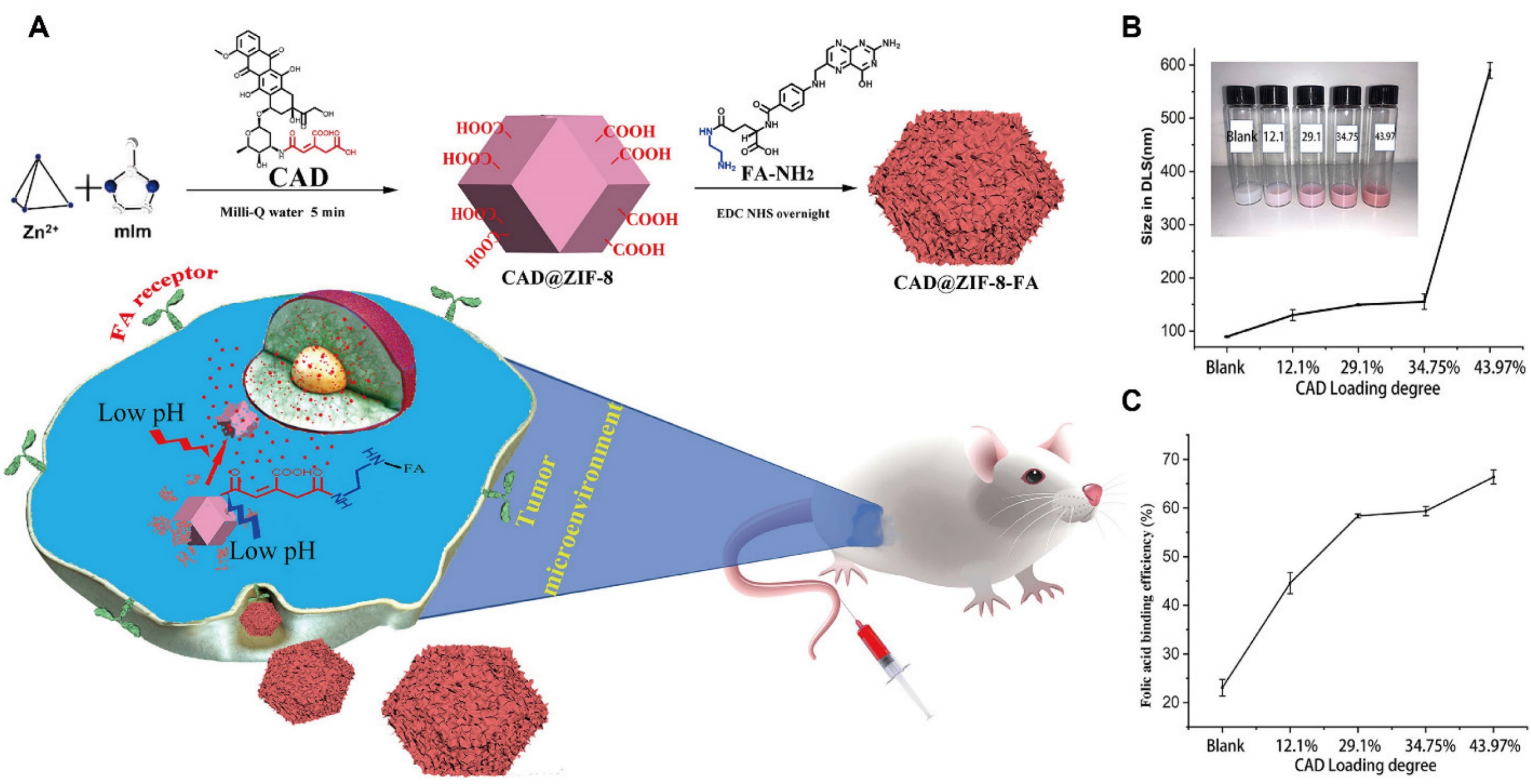
** Zn-MOFs for delivery CAD toward TME. (A)** Scheme of CAD@ZIF-8-FA NPs as a versatile nanocarrier for cancer treatment; **(B)** The size results of carriers with different drug loading rates in DLS; **(C)** The results of FA binding efficiency with different drug loading rates. Adapted with permission from 102, copyright 2020 American Chemical Society.

Zinc-based MOFs, particularly the ZIF series, have emerged as a major focus in DDS research since their initial report in 2006 97. Among them, ZIF-8 has attracted considerable attention due to its high stability under neutral and alkaline conditions, coupled with rapid degradation in acidic environments, which are particularly advantageous for tumor-targeted drug release. For instance, 5-fluorouracil (5-FU), a thymidylate synthase inhibitor widely used in cancer therapy, was successfully encapsulated within ZIF-8 with a loading capacity of 45.4%. This formulation exhibited markedly accelerated drug release at pH 5.0 compared with at pH 7.4, achieving 90% release within 12 h under acidic conditions 98. The first report of DOX loading into ZIF-8 in 2012 demonstrated a loading efficiency of 4.67% 99. Subsequent studies employing chemical surface modifications substantially improved DOX loading efficiencies and enhanced both tumor-targeting capability and pH-responsive release (Figure 5A) 102.

Further optimization was achieved through the incorporation of pH-sensitive linkers, namely cis-aconitic anhydride (CAA) conjugated to DOX, in combination with FA functionalization. This strategy enhanced drug-loading efficiency and cellular targeting, while the tuning of particle size offered additional control over release behavior (Figure 5B, C). Beyond DOX and 5-FU, ZIF-8 has also been widely utilized to encapsulate a variety of therapeutic agents, such as rapamycin 103, camptothecin 104 and caffeine 105, underscoring its versatility as a robust platform for pH-responsive drug delivery.

Polymetallic MOFs often exhibit enhanced drug delivery performance due to synergistic interactions among distinct metal centers. For example, Fe-Zn-ZIF-8 magnetic nanocarriers integrated the high drug-loading capacity of Fe-based MOFs with the pH-responsive behavior of Zn-MOFs, thereby achieving dual responsiveness to the TME 106. Similarly, bimetallic NiCo-MOFs doped with Tb^3+^ demonstrated high drug-loading efficiency, pH sensitivity, strong fluorescence emission, and imaging capability 107. Distinct metal centers impart unique physicochemical properties to MOFs: Fe-MOFs generally possess high porosity, Zn-MOFs offer great pH responsiveness, and Gd- and Mn-based MOFs offer superior imaging functionalities. Despite their compositional diversity, several consistent trends have emerged across MOF-based DDS research. First, MOFs generally exhibit substantial drug-loading capacities, often comparable to or exceeding those of conventional delivery platforms. Second, the internal pore structure of MOFs plays a critical role in determining drug encapsulation efficiency. Third, the hydrophilic-hydrophobic balance of organic linkers strongly influences drug-release kinetics, with more hydrophobic frameworks typically affording prolonged release profiles. Finally, the nature of the incorporated metal ions significantly affects biocompatibility; Fe-based MOFs generally exhibit lower toxicity than the Cr- or Zn-based analogues.

### MOFs for gene delivery

Over the past two decades, RNA- and DNA-based cancer therapies have attracted considerable attention as promising strategies for cancer treatment. However, as large biological macromolecules, nucleic acid therapeutics face substantial barriers to effective delivery, including high susceptibility to nuclease-mediated degradation in bloodstream, limited accumulation in tumor tissues, poor cellular uptake and poor endosomal escape. Gene delivery largely relies on nanocarriers, including cationic nanomaterials such as liposomes, polymeric vectors, and inorganic nanoparticles. Nonetheless, conventional nanocarriers often suffer from low transfection efficiency and may induce adverse effects, including hemolysis.

Gene therapy was initially conceived to correct diseases caused by defective or aberrant genes through the introduction of functional genes into target cells. More broadly, it now encompasses DNA- and RNA-based therapeutic approaches for treating diverse diseases, including ocular, cardiovascular, and oncological disorders. For cancer gene therapy, the delivery of naked nucleic acids is particularly challenging, as effective *in vivo* delivery must overcome multiple biological barriers to reach specific tissues. Moreover, therapeutic efficacy depends on intracellular delivery, which is hindered by electrostatic repulsion between negatively charged nucleic acids and cell membranes 108, as well as susceptibility to hydrolysis and enzymatic degradation. Therefore, the development of tailored delivery systems is essential to achieve targeted and efficient gene delivery.

### MOFs for DNA delivery

**Figure 6:**
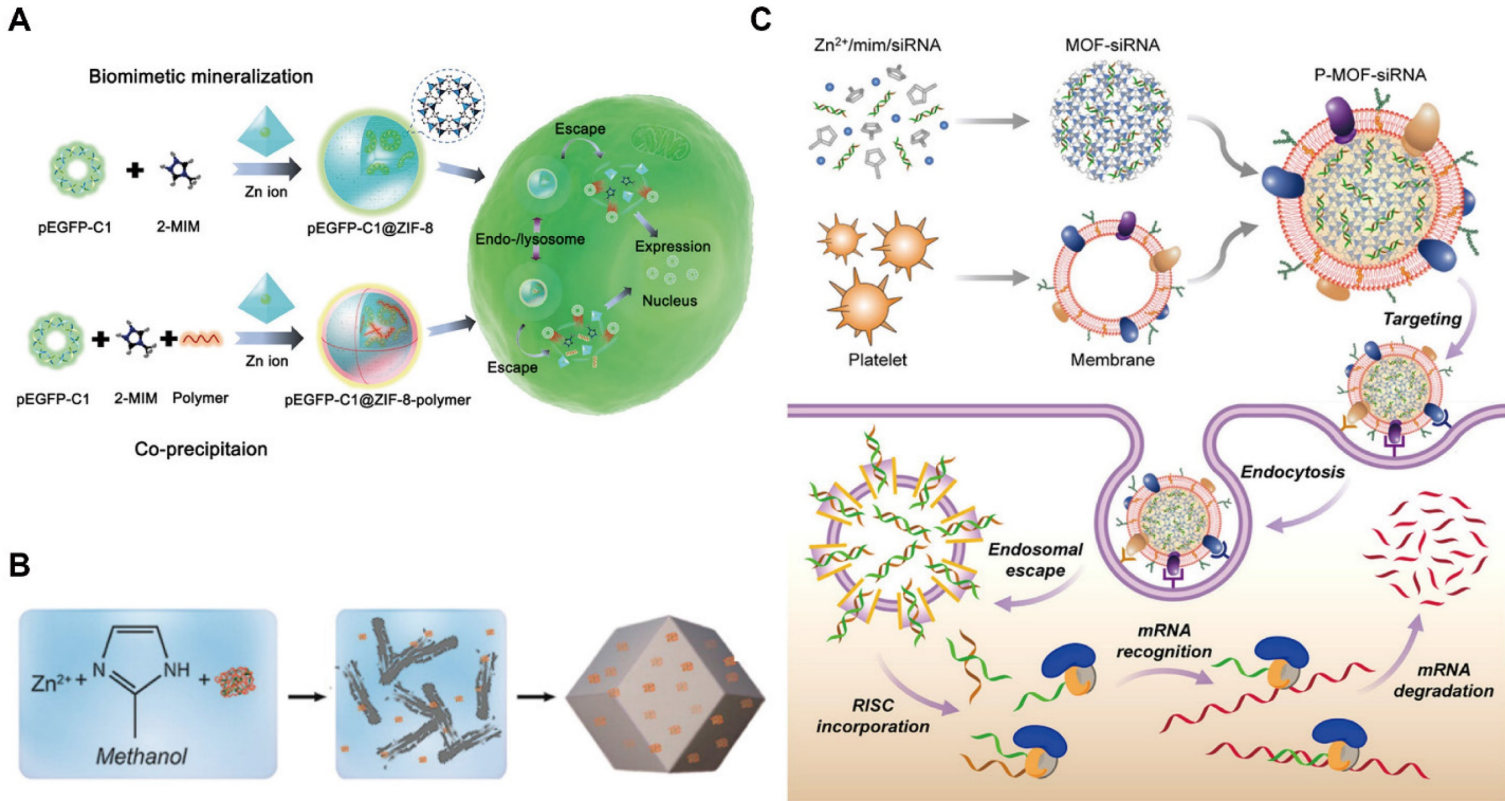
** (A)** Schematic representation of synthesis of pEGFP-C1@ZIF-8/pEGFP-C1@ZIF-8-polymer. Adapted with permission from 110, copyright 2019 WILEY-VCH; **(B)** The schematic shows the preparation of ZIF-8-based gene DDS 111, copyright 2014 American Chemical Society; **(C)** P-MOF-siRNAs (Platelet membrane-coated siRNA-loaded MOFs) for gene silencing. Adapted with permission from 112, copyright 2019 AAAS.

MOFs have emerged as promising platforms for gene delivery. To protect DNA from degradation and facilitate intracellular delivery, MOFs can be synthesized and loaded with DNA. For example, UiO-66-N_3_ (Zr_6_O_4_OH_4_(C_8_H_3_O_4_-N_3_)_6_) was surface-functionalized with oligonucleotides via a strain-promoted click reaction, representing the first MOF-nucleic acid conjugate 109. In this system, dibenzylcyclooctyne-modified DNA was conjugated to azide-functionalized UiO-66-N_3_. MOFs can also be surface-modified with DNA to create nano-composites, such as ZIF-67-based constructs, which exhibit favorable biocompatibility and sustained drug release. Although DNA-loaded MOFs effectively protect nucleic acids during transport, achieving tumor-specific delivery remains a significant challenge. To cope with this limitation, disulfide bonds have been incorporated into the loop region of DNA hairpins, enabling electrostatic and coordination interactions with MOFs and allowing cancer cell-specific release triggered by elevated endogenous glutathione (GSH) at tumor tissues. In addition, plasmid DNA (pDNA), including sequences encoding green fluorescent protein, has been loaded into ZIF-8 110. In this system, MOFs not only shielded DNA but also enhanced cellular uptake and promoted endosomal escape, resulting in efficient intracellular gene expression across multiple cell types (Figure 6A).

### MOFs for RNA delivery

RNA interference (RNAi) has been widely employed to selectively silence target messenger RNA (mRNA), thereby reducing gene and protein expression in gene therapy. Among RNAi therapeutics, synthetic siRNA and microRNA (miRNA) are the most extensively studied. Zeolitic imidazolate framework-8 (ZIF-8), a representative zinc-based MOF, exemplifies the potential of MOFs for RNA delivery (Figure 6B) 111. For instance, a light-responsive nanoswitch based on ZIF-8 enabled intracellular and lysosomal disruption-triggered gene release. When co-loaded with indocyanine green (ICG) and siRNA, ZIF-8 generated heat upon laser irradiation, promoting siRNA release into the cytoplasm and facilitating RNAi for cancer therapy. MOFs for siRNA delivery can also be fabricated via biomimetic synthesis strategies (Figure 6C). Cell membrane-coated MOFs have been developed to improve biocompatibility, immune evasion, and tumor targeting, thereby enhancing the translational potential of nucleic acid-based therapies. Similarly, ZIF-8 has been shown to protect miR-34a-m from *in vivo* degradation 112, allowing it to bind to complementary mRNA, suppress translation, and induce apoptosis. Compared with siRNA and miRNA, mRNA therapeutics face additional challenges due to their larger size and increased instability. To overcome these limitations, Zr-based MOFs chemically modified with polycationic ethanolamine-conjugated poly(glycidyl methacrylate) [PGMA(EA)] have been developed, these modified MOFs enhanced mRNA stability, cellular uptake and intracellular gene expression 113.

Overall, gene therapy has demonstrated broad potential for cancer therapy that are not fully addressable by traditional treatments. Nevertheless, several obstacles remain, including optimal target selection, formulation optimization, and carrier-cargo stability. Ideally, gene therapy should enable precise delivery, effectively suppress tumor growth, and minimize off-target effects. Recent advances in MOF-based gene delivery systems indicate substantial progress toward these goals (Table 3).

### MOFs for imaging agents

Molecular imaging enables *in vivo* visualization and analysis of cellular, molecular, and genetic processes, facilitating early, sensitive, and quantitative disease diagnosis. Molecular imaging has become an indispensable tool across a broad range of biomedical research applications by integrating principles from biology, physics, chemistry, and medicine. However, traditional small-molecule imaging agents often suffer from limited specificity and suboptimal signal sensitivity. Nanomaterials with precisely engineered sizes, shapes, and compositions offer enhanced imaging sensitivity and targeting specificity, prompting the development of advanced imaging vectors over the past decade to improve diagnostic accuracy 117,118.

MOFs have emerged as promising nanoplatforms for molecular imaging due to their intrinsic luminescence, tunable size and morphology, and selective adsorption properties. The high porosity, periodic framework structure, and abundant functional groups of MOFs provide numerous active sites for the conjugation of imaging agents, resulting in high loading capacity. MOFs can serve either as standalone imaging probes or as versatile carriers for integrating multiple imaging agents, enabling multimodal imaging and improving diagnostic precision. Moreover, MOFs constructed from intrinsically fluorescent metal nodes or organic ligands can function as autonomous imaging reagents. As summarized in Table 4, this section highlights recent advances in applications of MOFs for cancer imaging and theranostics, including infrared-photothermal (IP), FL, MRI, CT, PET, and PA imaging.

### MOFs for fluorescence imaging

**Figure 7:**
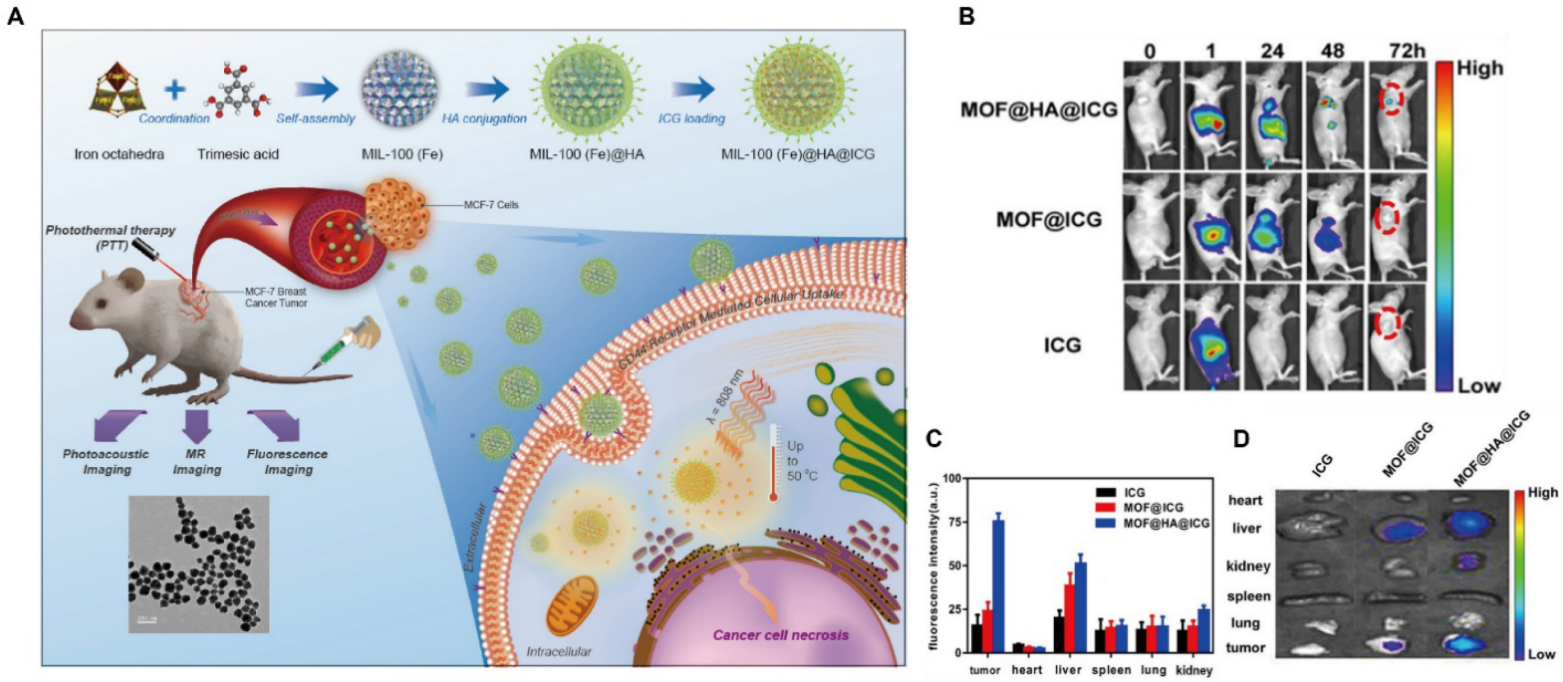
**Fluorescence imaging of MIL-100-Fe@HA@ICG. (A)** Schematic diagram of Fe-MOF synthesis and theranostics; **(B)** FL images of MCF-7 tumor-bearing mice injected with different treatments; **(C)** and **(D)** FL images and intensity of major organs and tumors. Adapted with permission from 119. copyright 2017 American Chemical Society.

Fluorescence imaging employs photo-luminescent probes to selectively target cancer cells, enabling direct *in vivo* visualization of the dynamic behavior of therapeutic agents. Over the past decades, fluorescent dyes, particularly ICG, have been widely used as imaging agents and approved by the Food and Drug Administration (FDA) for clinical applications. However, their use in cancer diagnostics is limited by poor aqueous solubility and low tumor specificity. MOFs offer an effective strategy to overcome these limitations. For instance, ICG-loaded MIL-100-Fe MOF nanoprobes were synthesized via HA surface modification (Figure 7A), exhibiting a uniform spherical morphology by transmission electron microscope (TEM). These nanoprobes achieved an ICG loading efficiency of up to 40%, leading to enhanced active tumor targeting and imaging intensity. The HA coating enabled active tumor targeting through specific recognition of CD44 receptors overexpressed on cancer cells.

Compared with free ICG, MOF@HA@ICG exhibited markedly higher fluorescence intensity at tumor sites, reduced degradation, and prolonged retention *in vivo*, with detectable signals persisting for up to 72 h post-administration (Figure 7B-D). These results highlight the excellent biocompatibility and biodegradability of the MOF-based system. Beyond cargo loading, MOFs can also serve as intrinsic fluorescent probes through the incorporation of emissive organic ligands. Porphyrins, characterized by favorable photostability, high fluorescence quantum yield, large Stokes shifts, and long excitation/emission wavelengths (λ_ex_=420 nm, λ_em_=660 nm), are particularly suitable for fluorescence imaging applications. For example, porphyrin-based MOFs coordinated with Fe³⁺ centers were loaded with dihydroartemisinin (DHA) to suppress premature drug release and subsequently coated with CaCO_3_ to yield NMOF@DHA@CaCO_3_. These MOF-based nanostructures functioned simultaneously as PSs and fluorescent probes 120.

### MOFs for magnetic resonance imaging

MRI is a non-invasive diagnostic technique based on the energy transitions of atomic nuclei possessing magnetic moments in an external magnetic field, with signal detection primarily arising from hydrogen nuclei in biological tissues. The acquired MRI signals are reconstructed into images; however, intrinsic tissue contrast is often limited because many tissues exhibit similar signal intensities, necessitating the use of contrast agents. These agents can be broadly categorized as T_1_ (positive) or T_2_ (negative) agents. T_1_ agents, such as paramagnetic Gd^3+^ or Mn^2+^ ions, shorten longitudinal relaxation times and enhance anatomical contrast, whereas T_2_ agents, typically superparamagnetic iron oxide nanoparticles, reduce transverse relaxation time to enhance image contrast, making areas of tissue damage or pathology more conspicuous. The rational selection of metal ions and organic ligands is critical, as coordination within complexes enhances thermodynamic stability and reduces toxicity compared to free ions 121. MOFs emerged as effective nanoplatforms in delivering paramagnetic ions for MRI. For instance, Gd³⁺-based MOFs synthesized via reverse microemulsion methods have demonstrated dual T_1_ and T_2_ contrast capabilities. Gd-MOFs loaded with anti-Programmed Death-1 (aPD-1) antibodies can integrate microwave hyperthermia, immunotherapy, and MRI, with tumor targeting enhanced by SCC7 membrane vesicle modification 122.

**Figure 8:**
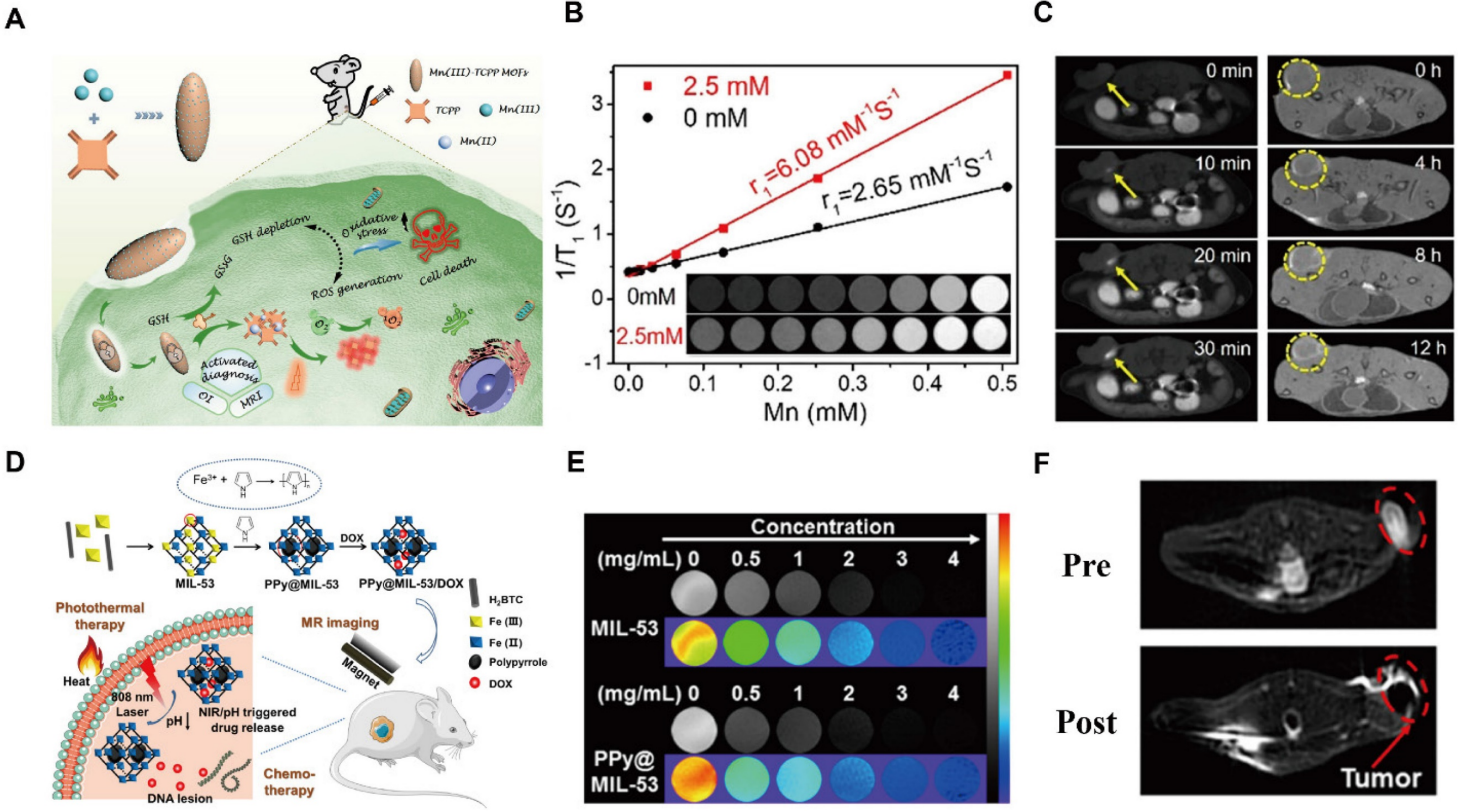
**Mn-MOF/Fe-MOF as T_1_/T_2_ contrast agents for MR imaging. (A)** Schematic illustration of a Mn(III)-MOF for imaging and therapy; **(B)** T_1_ imaging of MOFs with or without GSH; **(C)** T_1_ contrast signals after intratumoral injection (left) or intravenous injection (right). Adapted with permission from 124, copyright 2019 American Chemical Society; **(D)** Illustration of Fe-MOF synthesis for theranostics; **(E)** T_2_-weighted MRI images and pseudo-color imaging of MIL-53 and PPy@MIL-53; **(F)** T_2_-weighted MRI of PPy@MIL-53 (4T1 tumor lesion delineated by red lines). Adapted with permission from 128, copyright 2018 American Chemical Society.

Mn^2+^-based MOFs have attracted increasing attention due to their lower toxicity relative to Gd^3+^. Mn^2+^ and DOX co-loaded Zr-MOFs with PEGylation showed improved stability and evaded the reticuloendothelial system, and enabled T_1_-weighted MRI for cancer detection 123. Recent advances have also focused on Mn^3+^-centered MOFs, such as porphyrin-based Mn-MOFs, which exhibit enhanced stability relative to Mn^2+^ complexes. These GSH-activated nanosystems enable T_1_-weighted imaging by consuming tumor-site GSH and releasing Mn^2+^ contrast agents upon intracellular reduction (Figure 8A) 124. Mn-based MOFs commonly incorporate Mn^3+^ centers, electron-donating substituents on the porphyrin backbone further enhance the stability of Mn^3+^-MOFs. Upon cellular internalization, Mn^3+^-sealed MOF nanosystem deplete intracellular GSH, triggering the reduction of Mn-TCPP and subsequent Mn²⁺ release, thereby activating MRI contrast. *In vivo* fluorescence imaging and *ex vivo* organ analysis confirmed efficient tumor accumulation of the MOF probe (Figure 8B, C). Manganese oxides can also act as effective GSH scavengers. MnO_2_ shells coating drug-loaded Zr-MOFs imparted acid responsiveness and eliminated excess intratumoral GSH, while modification with 4T1 cell membranes further enhanced tumor targeting efficiency 125. In addition, doping MnO_x_ into the Zr-MOF shell via redox reactions combined with polymer modification yielded multifunctional, stimuli-responsive MOFs with MRI capability 126.

As representative T₂-type contrast agents, superparamagnetic iron oxide nanoparticles (SPIONs) have been extensively investigated. Although Fe-MOF-based MRI contrast agents exhibit improved biocompatibility, their relatively moderate relaxation rates limit imaging sensitivity and impede clinical translation. To overcome this challenge, integrating magnetic nanoparticles within MOF frameworks offers a promising strategy. For instance, a multifunctional core-shell nanoplatform, Fe_3_O_4_@UiO-66@WP6, was developed by incorporating Fe_3_O_4_@MOF with pillar[6]arene nanovalves 127. This system enables controlled multi-stimuli-responsive drug release and achieves MRI-guided cancer therapy. Release is accelerated under acidic conditions, while abnormal Zn^2+^ or Ca^2+^ levels associated with pathological states further modulate release behavior, effectively integrating intelligent drug delivery, MRI imaging, and chemotherapy within a single nanoplatform.

The MIL family, including MIL-53, MIL-100, and MIL-101, is well recognized for its high drug-loading capacity, controlled release behavior, T_2_-weighted MRI capability, and low toxicity. MIL-53, featuring mixed-valence iron centers, can function as a microreactor that provides unsaturated iron sites essential for pyrrole (Py) oxidation to polypyrrole (PPy), enabling *in situ* synthesis of a photothermal-chemotherapeutic MOF 128. Owing to its iron content, MIL-53 also serves as a T_2_-weighted MRI contrast agent (Figure 8D), allowing visualization of nanocomposite distribution and synergistic photothermal and chemotherapeutic effects. Its MRI performance was validated through *in vitro* T₂-weighted and pseudocolor imaging at various concentrations of MIL-53 and PPy@MIL-53, as well as *in vivo* MRI evaluation (Figure 8E, F). MIL-100 has likewise been employed to construct a core-shell MOF nanoplatforms with high drug-loading efficiency, primarily driven by hydrophobic interactions 129.

The system serves as a dual-mode MRI contrast agent for T_1_ (Mn)-T_2_ (Fe) imaging, exhibiting enhanced performance compared with single-mode agents. The enhanced r₁ relaxivity likely arose from amplified T_1_ effects induced by the external T_2_ component, while integrating T_1_ and T_2_ contrast agents mitigates spin coupling among T_2_ agents, reducing local magnetic field attenuation. Multimodal imaging strategies have been pursued via integrating different imaging modalities. A core-shell nanostructure, UCNP@Fe-MIL-101-NH_2_, was constructed using MIL-101 as the substrate 130. Subsequent surface modification with PEG and FA improved tumor targeting through FA receptor recognition. Both *in vitro* and *in vivo* studies demonstrated strong upconversion luminescence (UCL) and progressively enhanced T_2_-weighted MRI contrast in tumor regions. Overall, MOF-based multifunctional contrast agents, characterized by low toxicity, structural tunability, and integrated imaging capabilities, represent a promising nanoplatform for biomedical imaging and theranostic applications.

### MOFs for CT imaging

CT serves as an important complementary diagnostic modality that utilizes X-ray radiation to generate high-resolution three-dimensional images based on the differential attenuation of X-ray by tissues and organs. Conventional CT contrast agents are typically composed of high atomic number (Z) elements, such as iodine, barium, and bismuth, which effectively absorb X-rays. Representative clinical agents include iodixanol, barium sulfate, and gadopentetate dimeglumine.

**Figure 9:**
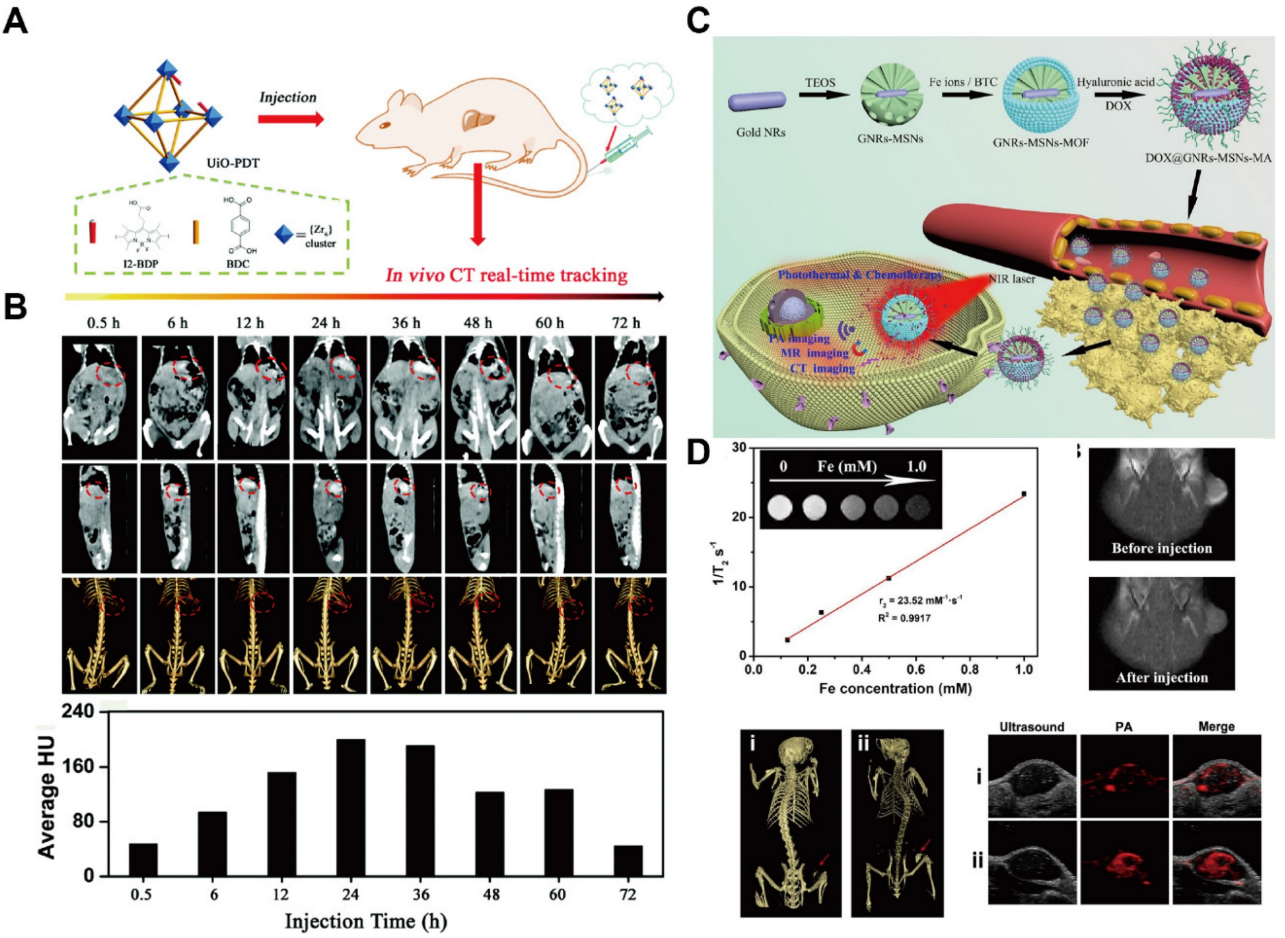
** MOFs for tumor imaging by CT. (A)** Synthesis of UiO-PDT MOF for CT imaging; (B) CT images of Walker-256 tumors in a rat by UiO-PDT MOFs. Adapted with permission from 132, copyright © 2020 Royal Society of Chemistry; (C) Diagrammatic representation of the synthesis, imaging, and therapy of the GNRs-MSNs-MA; (D) MRI/CT/PA images of 4T1 tumors in mice with (i) GNRs-MSNs-MOF and (ii) GNRs-MSNs-MA. Adapted with permission from 133, copyright © 2021 Elsevier.

MOFs incorporating high-Z elements have demonstrated intrinsic CT imaging capability and have offered a promising nanoplatform as CT contrast agents. In 2009, a class of MOFs with CT contrast potential was synthesized using Cu^2+^ and Zn^2+^ as metal centers in combination with an iodide-based organic ligand (Z_I_ = 53) 131. The theoretical iodine loadings of these MOFs reached 63.2% and 55.3%, respectively, both exceeding that of iodixanol (49%)—highlighting their potential for enhanced CT contrast. Moreover, a highly crystalline and monodisperse UiO-PDT nanocrystal was fabricated by incorporating a photoactive iodine-BODIPY dye ligand into UiO-type MOFs (Figure 9A) 132. *In vivo* studies at a dose of 100 mg kg^-1^ demonstrated negligible acute and subacute toxicity, with no significant adverse effects observed. UiO-PDT exhibited excellent CT imaging capacities, achieving optimal contrast enhancement at 24 h post intravenous administration (Figure 9B).

Gold (Au) possesses superior X-ray attenuation properties owing to its high atomic number (Z_Au_ = 79) and K-edge energy (k_Au_ = 81), providing stronger contrast than iodine-based CT agents. Gold nanorods (GNRs) encapsulated within ZIF-8 have been developed as dual-functional CT contrast and photothermal agents, with DOX further loaded following lactobionic acid (LA) modification to enable liver cancer targeting 161. The resultant near-infrared (NIR)/pH-responsive MOF system allows for CT-guided chemotherapy and photothermal therapy (PTT) combinational therapy. Owing to their high drug-loading capacity, MOFs can also be integrated with other nanoparticles to enhance CT contrast and enable multimodal imaging. For example, GNRs encapsulated within MOFs have demonstrated effective CT imaging capability (Figure 9C) 133. In this design, an iron-based MOF was synthesized *in situ* on dendritic mesoporous silica-coated GNRs (GNRs-MSN) to form a core-shell nanostructure, followed by HA modification to improve tumor targeting. The strong X-ray attenuation of GNRs embedded within the MOF confirmed their CT contrast performance. Moreover, the resultant nanoplatform exhibited tri-modal imaging capability (MRI/CT/PA) and enhanced tumor accumulation after HA modification (Figure 9D). Nevertheless, the complexity of the synthesis process remains a major challenge, limiting large-scale production and clinical translation. Current research efforts are therefore focused on simplifying MOF fabrication while further improving their multimodal imaging performance.

### MOFs for PET imaging

**Figure 10:**
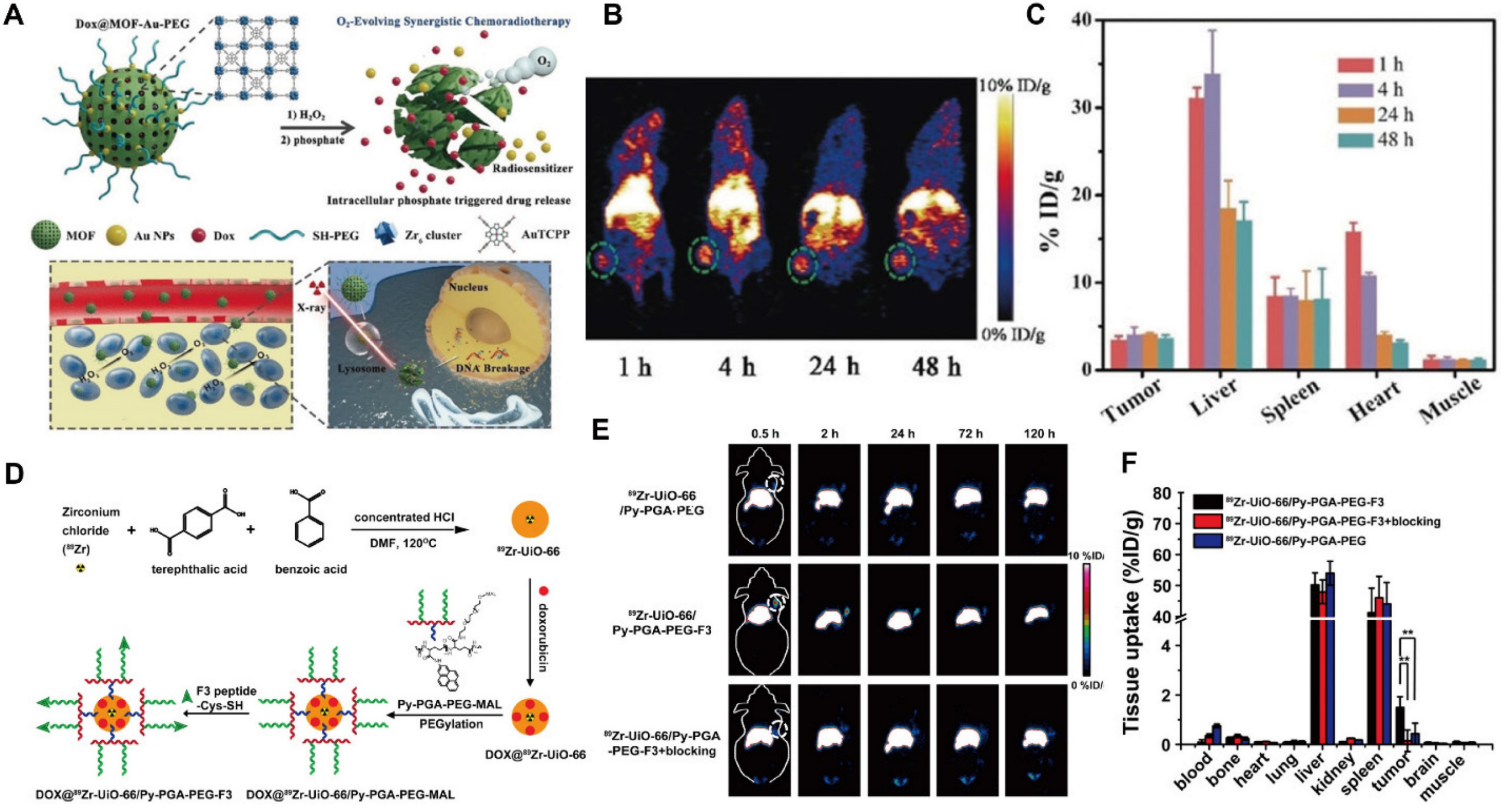
** MOFs for tumor imaging by PET. (A)** Schematic representation of DOX@MOF-Au-PEG synergistic chemoradiotherapy; **(B)** PET imaging of ^64^Cu-MOF-Au-PEG; **(C)** Quantitative ROI assay of major tissues. Adapted with permission from 135, copyright 2019 WILEY-VCH; **(D)** Schematic of the synthesis of ^89^Zr-UiO-66/Py-PGA-PEG-F3 Conjugates; **(E)** PET images of MDA-MB-231 tumors in mice by ^89^Zr-MOFs; **(F)** Organ distribution of ^89^Zr-MOFs at 120 h. Adapted with permission from 136, copyright 2017 American Chemical Society.

PET is a widely used nuclear imaging modality that provides tissue-, cellular-, and molecular-level information by detecting positrons emitted from short-lived radioisotopes such as ^11^C, ^13^N, ^15^O, ^18^F, ^64^Cu, ^68^Ga, ^89^Zr, and ^124^I. Despite its high sensitivity, excellent quantitative capability and deep tissue penetration, PET imaging faces several challenges, notably the short half-lives of radionuclides and limited diagnostic specificity 134. MOFs, owing to their high drug-loading capacities and multiple metal-ion nodes, have emerged as promising platforms for PET imaging. For example, a Zr-TCPP MOF integrated with gold NPs, loaded with DOX, and further modified with PEG-SH was developed as an oxygen-regulating nanoplatform (Figure 10A) 135. Following intravenous injection of ^64^Cu-labeled MOF-Au-PEG in tumor-bearing mice, whole-body and organ-specific PET imaging revealed pronounced tumor accumulation at multiple time points (Figure 10B, C).

Beyond surface radiolabeling, the intrinsic structural versatility of MOFs allows direct incorporation of positron-emitting isotopes into their frameworks. For example, ^89^Zr was directly introduced into UiO-66 to construct a DOX-loaded radioactive MOF 136. Subsequent surface functionalization with pyrene-polyethylene glycol (Py-PGA-PEG) and an F3 peptide ligand endowed the system with enhanced targeting toward triple-negative breast cancer (Figure 10D). PET imaging demonstrated that tumor accumulation of the modified MOF was approximately three- to fourfold higher than that of the unmodified counterpart (Figure 10E). Comprehensive toxicity studies confirmed the absence of both acute and chronic toxicity. However, the susceptibility of Zr-based MOFs to degradation in phosphate-rich environments remains a limitation. To address this, bis[2-(methacryloxy)ethyl] phosphate (BMAP) ligands were grafted on the surface of ^64^Cu-Zr-MOFs, effectively preventing acid and phosphate-induced decomposition, prolonging circulation time, and enhancing targeted delivery 137. Upon reaching TME, intracellular GSH triggered polymer degradation, exposing the MOF core to phosphate ions and promoting the release of encapsulated therapeutics. Overall, the integration of stimuli-responsive designs with intrinsically radioactive MOFs represents a promising strategy to improve drug utilization efficiency and PET imaging performance (Figure 10F).

### MOFs for PA imaging

PA imaging is a rapidly advancing, non-invasive, and non-ionizing imaging modality that utilizes pulsed laser irradiation to generate US waves in light-absorbing regions of biological tissues. The resultant acoustic signals are detected by an external ultrasound transducer and reconstructed into high-contrast images of internal structures. By combining the advantages of optical excitation and ultrasonic detection, PA imaging offers high spatial resolution and relatively deep tissue penetration, making it highly promising for early cancer diagnosis and treatment monitoring 138.

**Figure 11:**
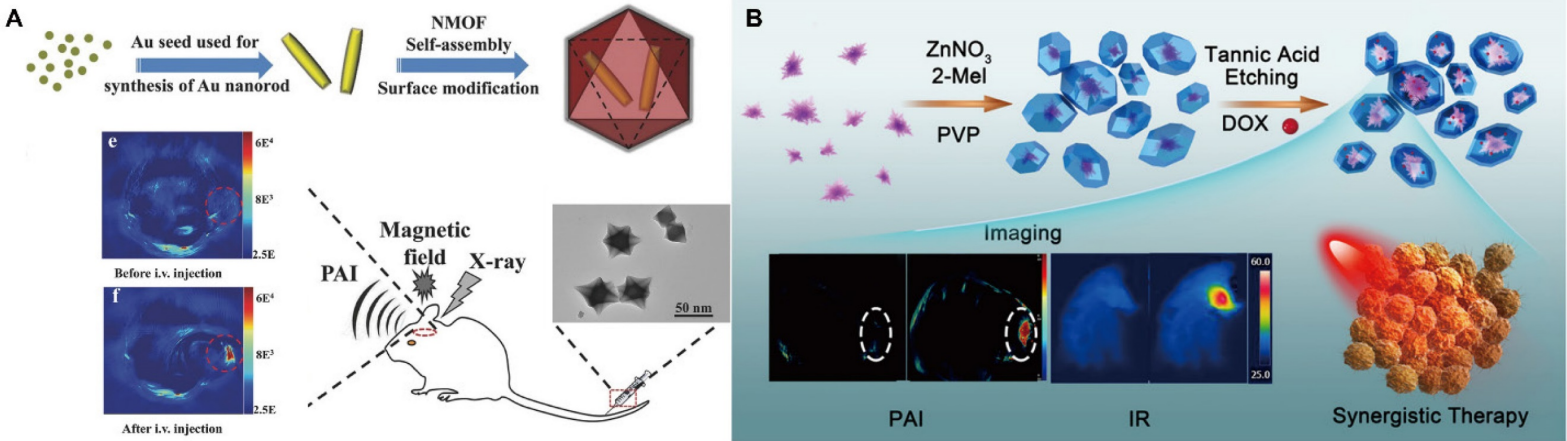
**MOFs for tumor imaging by PAI. (A)** Schematic illustration of the synthesis of core-shell Au@MIL-88(Fe) nanostars and one of the PAI images in triple-modal. Reproduced with permission. Adapted with permission from 140, copyright 2017 WILEY-VCH. **(B)** Schematic illustration of the fabrication of Au@MOF-DOX, PAI imaging effects. Adapted with permission from 141, copyright 2019 American Chemical Society.

Despite these advantages, PA imaging often suffers from weak intrinsic signal intensity in biological tissues, necessitating a high amount of exogenous contrast agents at the target site. However, the limited targeting ability of many conventional agents reduces their effective utilization. To address these challenges, MOFs have been explored as versatile PA contrast platforms capable of enhancing imaging sensitivity and therapeutic efficacy. For instance, embedding Au nanoparticles within Zn-MOFs enabled pH- and GSH-responsive release, inducing strong plasmonic NIR absorption and thereby enhancing PA signal intensity 139. Similarly, core-shell Au@MIL-88(A) nanostructures (89 nm) with star-like morphology exhibit high crystallinity and multimodal PAI/MRI/CT imaging capabilities (Figure 11A) 140. Moreover, the integration of MOFs with chemotherapeutics enables synergistic imaging and therapy. A yolk-shell nanostructure was fabricated by depositing ZIF-8 on Au nanostars, followed by tannic acid etching and DOX loading, resulting in a multifunctional platform that combines enhanced PA imaging with chemotherapy (Figure 11B) 141. In addition, chemical surface modification can endow MOFs with active targeting functionality, further improving PA imaging performance.

Although most MOFs lack intrinsic PA activity, their tunable composition, favorable biocompatibility, and high porous structure render them promising scaffolds for the development of next-generation PA contrast agents. Enhancing the sensitivity and selectivity of MOF-based agents remains a highly, and continued advances in rational design are expected to significantly broaden their applications in theranostics.

### MOFs for delivering other bioactive compounds

While immunotherapy has attracted considerable interest as a promising cancer treatment, its clinical efficacy remains limited by challenges such as low response rates, limited tumor specificity, and systemic toxicity. MOFs, owing to their high loading capacity and large surface area, have emerged as versatile platforms for enhancing antitumor immunotherapy. MOFs can potentiate cancer immunotherapy through multiple mechanisms, including targeted delivery of antigens and immunostimulatory agents, modulation of immune dysfunction within the TME, and synergistic therapeutic effects. For instance, ovalbumin (OVA) encapsulated in ZIF-8, with surface modification by unmethylated cytosine-phosphate-guanine oligo-deoxynucleotides (CpG ODNs), enables precise targeted immunotherapy, effectively promoting antigen presentation while enhancing the overall biocompatibility and immunogenicity 142. Similarly, incorporating the Toll-like receptor 7/8 (TLR7/8) agonist R848 into bimetallic FeMn-MOFs allows HA-mediated targeted delivery, where the released metal ions and R848 within the TME induce ICD and potentiate immunotherapy 143.

Given their catalytic versatility, enzymes have been explored for metabolic modulation in cancer therapy; however, their poor stability limits standalone application. MOFs provide a protective and stabilizing matrix for enzyme delivery. For example, lactate oxidase loaded into Fe-MOFs, in combination with siRNA, can deplete lactic acid and reverse the immunosuppressive TME 144. DNAzyme-MOFs systems further function as multifunctional nanocarriers capable of accommodating nucleic acids (*e.g.*, DNAzymes and CpG) through electrostatic and coordination interactions 145. The acidic TME facilitates ZIF-8 degradation, enabling controlled release of DNAzymes and photosensitizer Ce6 for combinational therapy. Moreover, MOFs possess intrinsic diagnostic and therapeutic potential, supporting their development as nanotheranostic agents that integrate tumor detection and treatment. The following section summarizes recent advances in MOF-based strategies across diverse cancer therapies.

## MOFs for cancer theranostics

Conventional cancer treatments, including surgery, chemotherapy, and RT, remain the mainstay of clinical oncology. However, chemotherapy is often limited by non-specific drug distribution, multidrug resistance, and severe side effects. To address these challenges, the integration of traditional therapeutics with nanomedicine has emerged as a promising strategy. Compared with conventional materials, MOFs offer distinct advantages for drug delivery. As discussed earlier, MOFs possess high drug-loading capacities and can be readily engineered for targeted and stimuli-responsive release. Consequently, multifunctional MOF-based nanoplatforms have demonstrated considerable potential and have been extensively explored for cancer therapy. MOFs can act as efficient carriers for therapeutic agents or imaging contrast agents, thereby enabling simultaneous treatment and diagnosis of tumors. Moreover, MOF-based systems have advanced conventional therapies and facilitated the development of emerging treatment modalities such as radiotherapy, PTT, PDT, chemodynamic therapy (CDT), sonodynamic therapy (SDT), and immunotherapy. As summarized in Table 5, this section focuses on multifunctional MOF-based nanoplatforms designed for integrated cancer therapy.

### MOFs for RT based theranostics

RT, a clinically available approach for cancer treatment, exerts its therapeutic purpose by utilizing ionizing radiation to inhibit cancer cells within the irradiated region. However, the intrinsic radio-resistance of certain tumor types, together with unavoidable radiation-induced toxicity to surrounding normal tissues, often limits the efficacy of RT as a standalone treatment. These challenges have motivated the development of radiosensitizers—agents with low intrinsic toxicity that can enhance tumor sensitivity to radiation. MOFs have been investigated as promising radiosensitizers for cancer radiotherapy.

**Figure 12:**
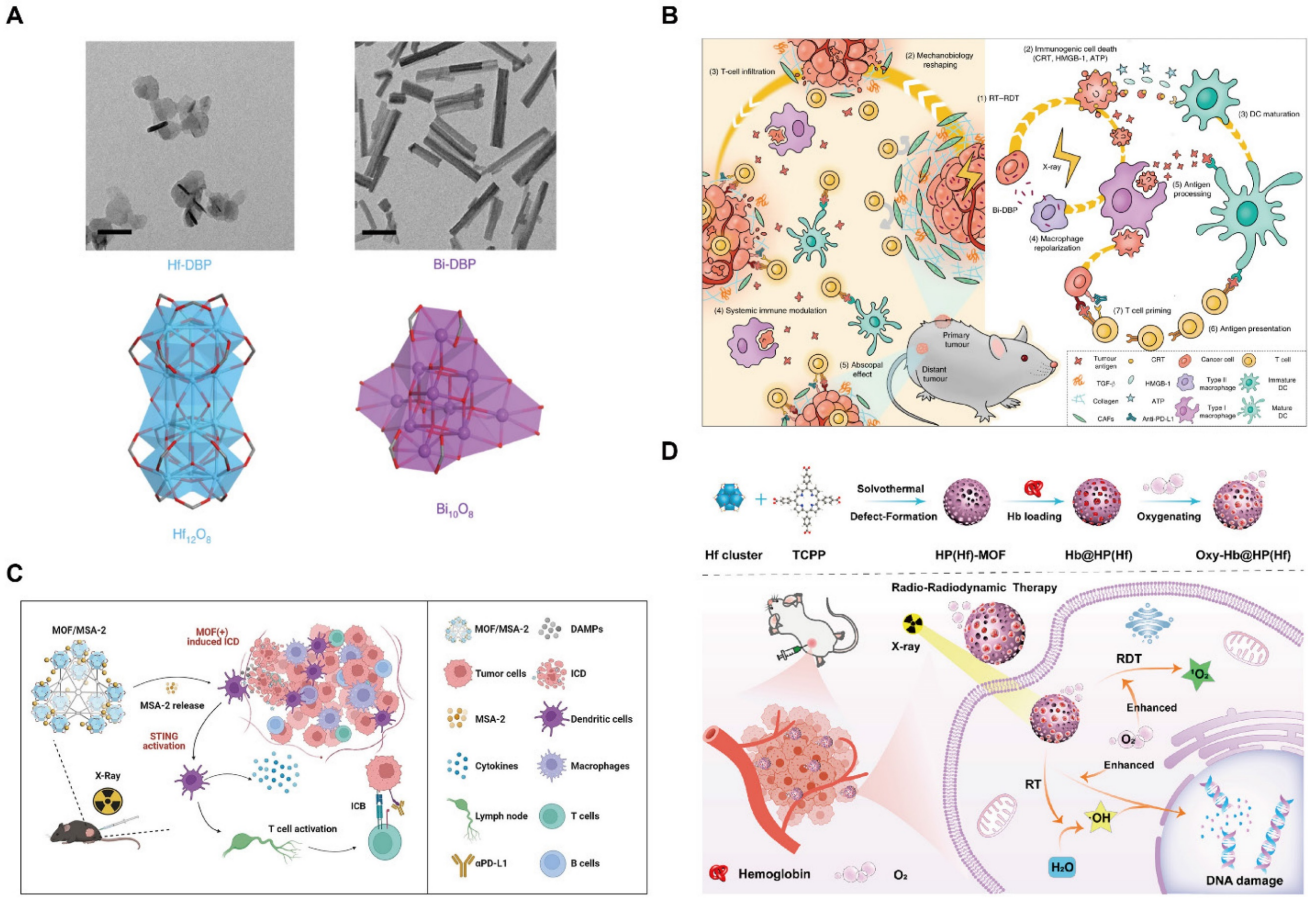
**(A)** Transmission electron microscopy images of Hf-DBP/Bi-DBP and structures of Hf_12_O_8_ SBUs in Hf-DBP and Bi10O8 SBUs in Bi-DBP (Scale bars: 100 nm); **(B)** Scheme of Bi-DBP-mediated RT-RDT for modulating biomechanics to promote T-cell infiltration. Adapted with permission from 146, copyright 2022 Springer Nature; **(C)** Synergistic mechanism of MOF/MSA-2 for radio-sensitization and immune activation. Adapted with permission from 147, copyright 2024 WILEY-VCH; **(D)** Schematic illustration of the oxygen-enriched Hb@HP(Hf) nanosensitizer for RT-RDT cancer therapy: synthesis and mechanism of action. Adapted with permission from 148, copyright 2023 American Chemical Society.

Hf-MOF and Zr-MOF, constructed from high-atomic-number metal centers within the UiO framework, have been shown to act as efficient X-ray absorbers, thereby enhancing RT therapeutic efficiency to some extent. Subsequently, metal elements with even higher atomic numbers (such as bismuth) were introduced into porphyrin-based MOFs (Figure 12A, B). Compared to Hf, these elements exhibit superior X-ray absorption and energy transfer efficiencies, resulting in more pronounced radiosensitization effect 146.

Beyond single-modality enhancement, current research increasingly focuses on RT-based multi-modal therapy strategies. For instance, leveraging the high drug-loading capability of MOFs, the stimulator of interferon genes (STING) agonist 4-(5,6-dimethoxybenzo[b]thiophen-2-yl)-4-oxobutanoic acid (MSA-2) has been encapsulated into a MOF based on Hf6 SBUs and hexakis(4'-carboxy[1,1'-biphenyl]-4-yl)benzene (HCBB) bridging ligands for robust cancer radio-immunotherapy (Figure 12C) 147. Alternatively, RT can be combined with radiodynamic therapy (RDT), in which scintillators convert X-ray radiation energy into visible light to activate photosensitizers. This process promoted the interactions between photosensitizers and oxygen to generate singlet oxygen (^1^O_2_), thereby inducing extensive tumor cell apoptosis. As an illustrative example, hemoglobin (Hb) was loaded on Hf-TCPP to provide a substantial oxygen supply, alleviating tumor hypoxia and significantly enhancing the therapeutic efficacy of the combined RT-RDT strategy (Figure 12D) 148.

### MOFs for PTT based theranostics

PTT, which eradicates tumors through localized thermal ablation, has been extensively investigated owing to its minimal side effects. Temperature elevation at the tumor site arises from the conversion of light energy into heat by photothermal agents, leading to direct hyperthermia in cancer cells. Upon accumulation at the lesion site, these agents are irradiated with NIR light to achieve effective tumor ablation. ICG, an FDA-approved NIR dye, is widely recognized as an efficient photothermal agent. For example, ZIF-8 loaded with ICG enabled fluorescence imaging-guided PTT, integrating diagnosis and therapy within a single platform 149. In addition, the incorporation of surface-modified polymers as photothermal agents, together with strategies to enhance the stability and biocompatibility of MOFs, represents effective approaches. PDA can be coated on the surface of ZIF-8 to function as a photothermal agent, while the MOF interior serves as a reservoir for the chemotherapeutic drug Methotrexate (MTX), enabling combinational photothermal and chemotherapy treatment 150.

**Figure 13:**
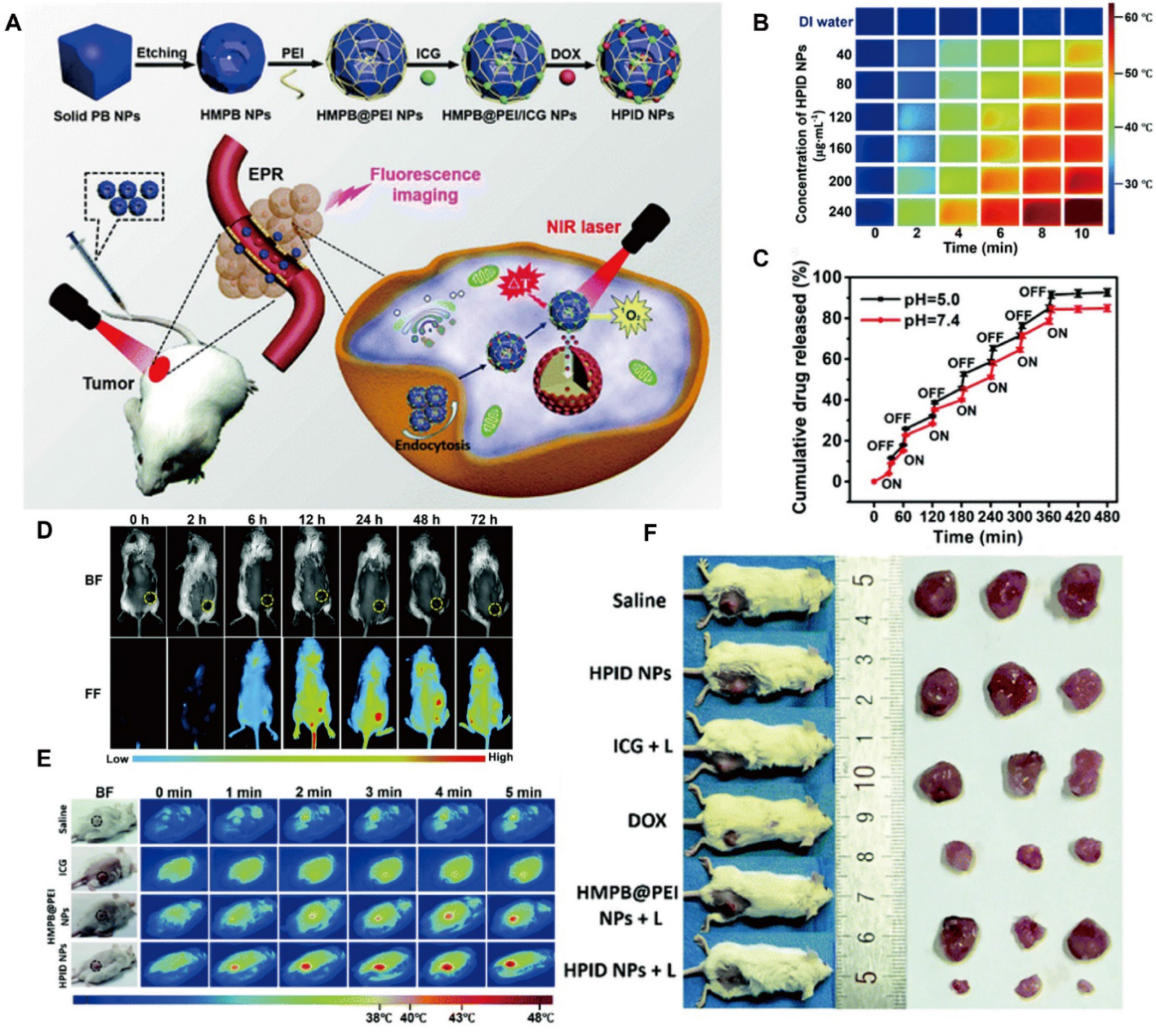
**(A)** Schematic diagram of the synthesis process of HPID NPs; **(B)** Infrared thermal images with HPID NPs (808 nm, 2 W cm^-2^); **(C)** DOX release kinetics of HPID NPs; **(D)** Bright-field and fluorescence images after *i.v.* of HPID NPs; **(E)** Thermal imaging of tumor site irradiated by NIR laser at different times. Adapted with permission from 151, copyright 2019 Royal Society of Chemistry.

Beyond serving as drug carriers, certain MOFs also exhibit intrinsic photothermal properties and can be applied to enhance the effect of conventional PTT agents. For example, a MOF can be etched to form hollow mesoporous nanoparticles (HMPB NPs) with improved drug-loading capacity 151. These nanoparticles can be co-loaded with ICG and DOX to generate HPID NPs (Figure 13A). The HPID NPs exhibit enhanced photothermal performance, while the MOF framework mediates temperature-responsive drug release (Figure 13B, C). Furthermore, the MOF-based system enabled the monitoring of drug accumulation and distribution via fluorescence and infrared imaging (Figure 13D, E), thereby integrating tumor diagnosis with PTT-based triple combination therapy (Figure 13F).

### MOFs for PDT based theranostics

As outlined earlier, PDT represents one of the three therapeutic modalities within the HPID nanomedical platform. PDT is a light-activated anticancer strategy that requires the concurrent presence of three components: a PS, light of a specific wavelength, and endogenous O_2_. The treatment process generally consists of two stages: targeted delivery of the PS, followed by controlled light irradiation. Upon activation by light, the PS transfers energy to surrounding O_2_ molecules, generating reactive oxygen species (ROS) that ultimately induce cancer cell death. PSs, such as hematoporphyrin derivatives, were first introduced in cancer PDT in the 1970s. However, the intrinsic hydrophobicity of many PSs has limited their clinical application. To address this limitation, various strategies have been developed to load PSs into MOFs. For instance, Ce6, 2-((4'-(2,2-bis(4-methoxyphenyl)-1-phenylvinyl)-[1,1'-biphenyl]-4-yl)(phenyl)methylene)malononitrile (TPEDC), and (E)-2-(4-(4-(2,2-bis(4-methoxyphenyl)-1-phenylvinyl)styryl)-3-cyano-5,5-dimethylfuran-2(5H)-ylidene)malononitrile (TPETCF) have been encapsulated in MIL-100-Fe and further modified with F127 to enhance tumor targeting, resulting in effective PDT 152. Encapsulation within MOFs prevents PS aggregation and premature leakage while minimizing undesired interaction with O_2_ prior to reaching the lesion site. Porphyrins can also serve as organic ligands for MOFs, giving rise to intrinsic photodynamic MOFs that inherently function as PSs. For example, 5,15-bis(p-benzoic acid) porphyrin (H2DBP) has been coordinated with Hf^4+^ SBUs to construct porphyrin-based MOFs for cancer PDT 44. This design strategy suppressed porphyrin self-quenching associated with aggregation and significantly enhanced PDT efficacy. Subsequently, numerous studies have reported the development of intrinsic photodynamic MOFs 153. Among these, Zr^4+^-based MOFs incorporating tetrakis(4-carb-oxyphenyl)porphyrin (TCPP) as the organic ligand have attracted particular attention due to their high ROS generation, excellent structural stability, and favorable biodegradability 154*.*

**Figure 14:**
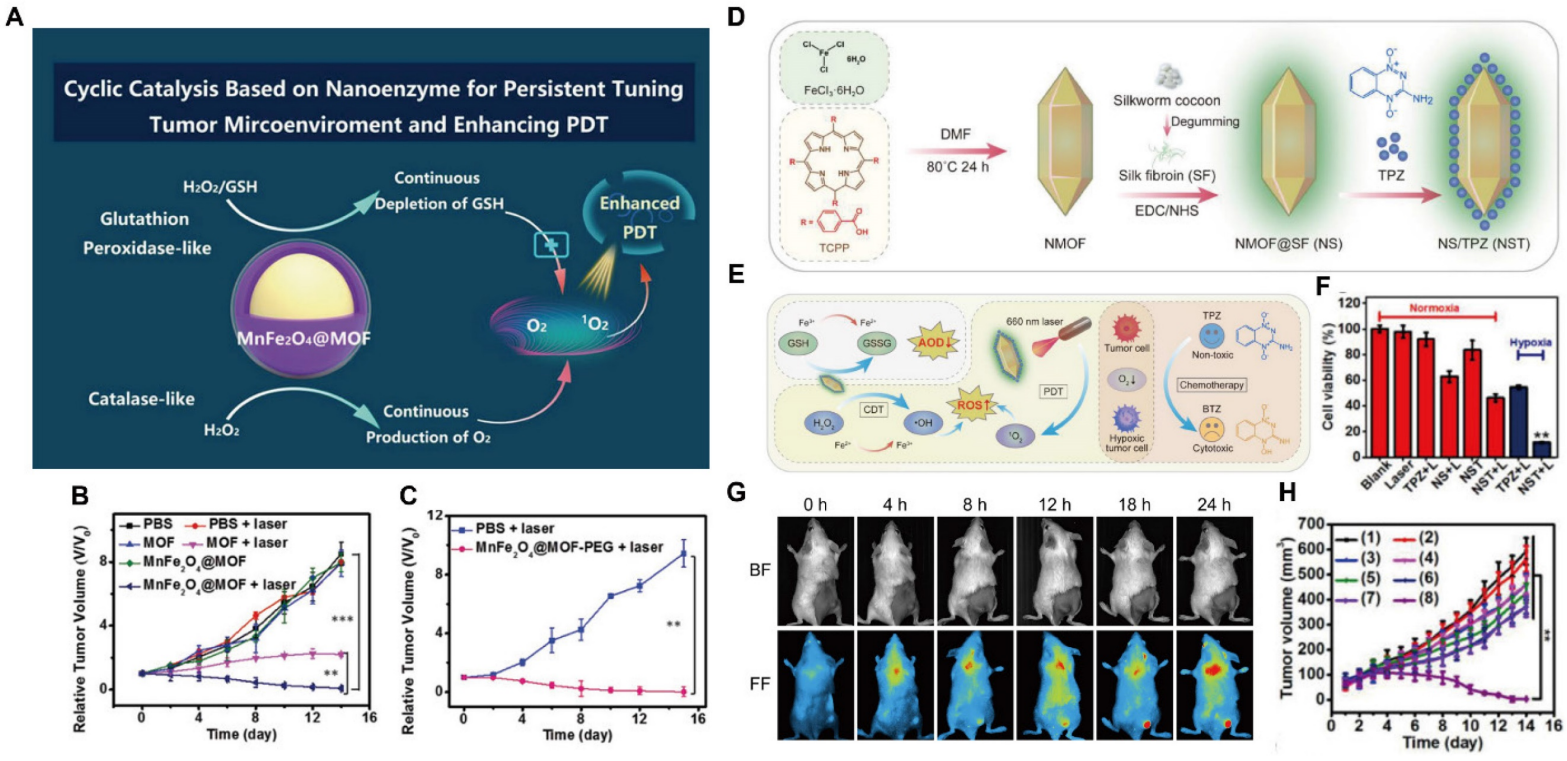
**(A)** The core-shell structure and mechanism of MnFe_2_O_4_@MOF for continuous depletion of GSH and production of O_2_ to enhance PDT; **(B)** Tumor growth curve after Intratumoral injection; **(C)** Intravenous injection. Adapted with permission from 157, copyright 2019 WILEY-VCH. **(D)** and **(E)** The structure and mechanism of treatment of NST; **(F)** Viability of tumor cells after various treatments under normoxic/hypoxic conditions; **(G)** Bright-field and fluorescence images after *i.v.* of NST NPs; **(H)** Changes in tumor volume. Adapted with permission from 158, copyright 2022 Elsevier.

The therapeutic efficacy of PDT can be further enhanced by optimizing ROS generation efficiency within TME. The most direct strategy involves increasing endogenous O_2_ levels and/or reducing ROS scavenging in the TME. One established approach is to load MnO_2_ into MOFs, where MnO_2_ decomposes the elevated hydrogen peroxide (H_2_O_2_) in the TME to generate O_2_, thereby alleviating local O_2_ concentration. Concurrently, MnO_2_ degrades into Mn^2+^ within the TME, enabling MRI-guided tumor diagnosis 155,156. Moreover, core-shell MOF nanostructures have been engineered to simultaneously convert H_2_O_2_ into O_2_ and deplete GSH, thereby minimizing ROS scavenging and achieving enhanced PDT efficacy (Figure 14A) 157. The release of Mn^2+^ enabled MRI-guided, precision cancer treatment (Figure 14B, C).The localized O_2_ depletion induced during PDT can also potentiate the efficacy of hypoxia-activated chemotherapeutic agents, providing a foundation for PDT-based combination therapy. Tirapazamine (TPZ), a hypoxia-activated prodrug (HAP), exhibits preferential cytotoxicity under hypoxic conditions. Silk fibroin-modified Fe-TCPP MOFs loaded with TPZ (denoted as NST) enabled simultaneous PDT and hypoxia-activated chemotherapy. Within the TME, MOF degradation and the Fe^3+^/Fe^2+^ redox cycle reduce ROS consumption by reacting with GSH, while further depleting endogenous O_2_ to enhance PDT efficacy and amplify HAP therapeutic effects (Figure 14D, E) 158. Fluorescence imaging confirmed tumor accumulation of NST. Both *in vitro* (Figure 14F, G) and *in vivo* studies (Figure 14H) demonstrating that the combinational treatment significantly outperformed single-modality therapies.

### MOFs for SDT based theranostics

Despite the development of diverse combination treatment strategies that have significantly enhanced PDT efficacy, its therapeutic potential remains fundamentally constrained by the limited penetration depth of light in biological tissues. Consequently, PDT exhibits reduced efficacy in the treatment of deep-seated solid tumors. To overcome this limitation, SDT has emerged as a promising non-invasive treatment modality. SDT employs a sonosensitizer and O_2_ to generate ROS, gas bubbles, and cavitation effects under low-intensity or dose-graded short-term repeated ultrasound irradiation. Due to its relatively higher tissue penetration, spatial selectivity and minimal off-target damage to surrounding healthy tissues, SDT exhibits an excellent safety profile and considerable potential. However, SDT faces two critical challenges. First, the bioavailability of sonosensitizers, particularly organic sonosensitizers, is often limited, and they typically lack effective tumor-targeting capabilities. Second, similar to PDT, the hypoxic tumor microenvironment restricts ROS generation and compromises SDT efficacy. Addressing these limitations by improving sonosensitizer bioavailability and endogenous O_2_ concentration is essential for fully realizing the therapeutic potential of SDT 159.

**Figure 15:**
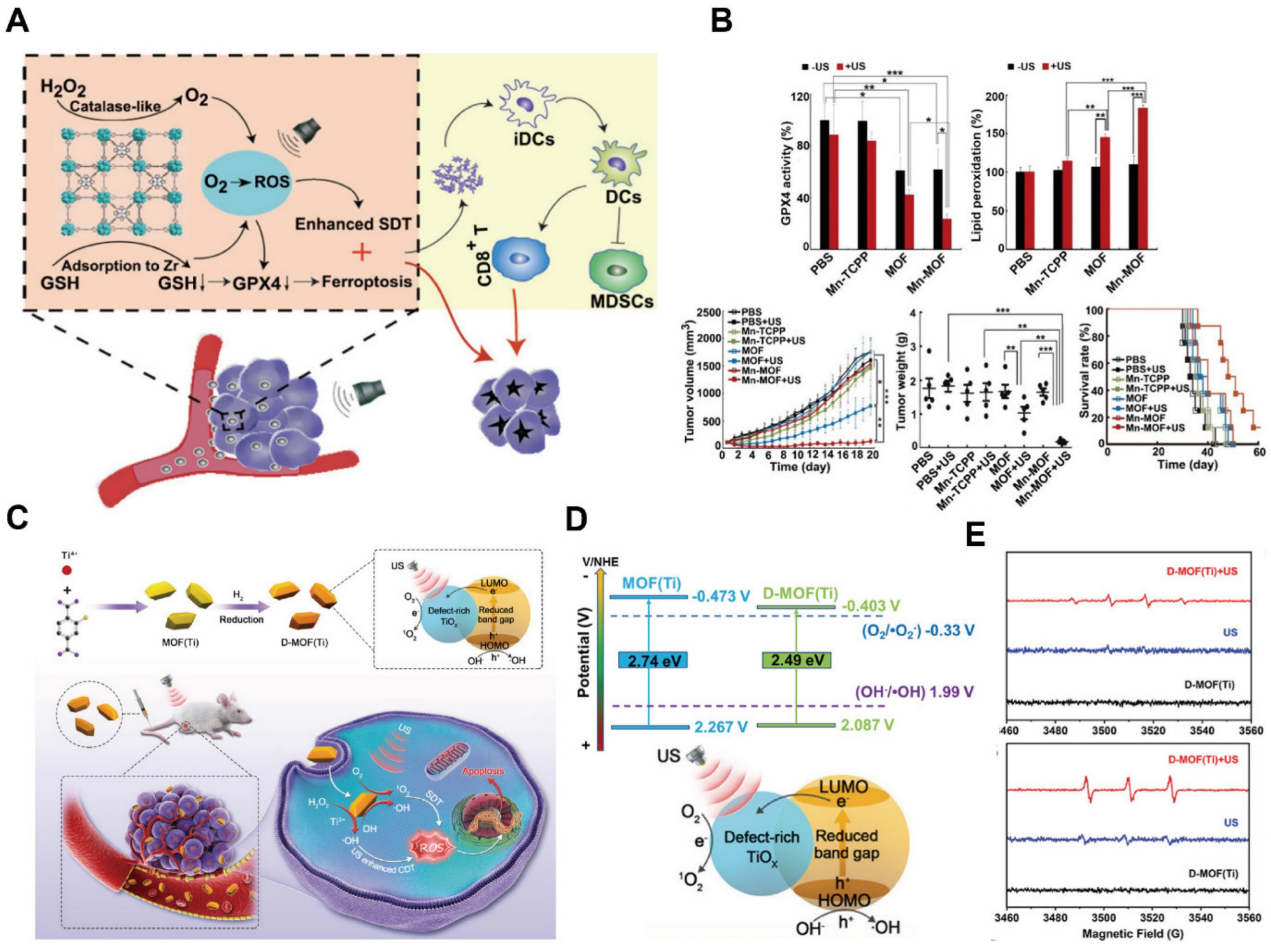
**(A)** Schematic diagram of Mn-MOF enhancing combination in cancer treatment (SDT and ferroptosis); **(B)** GPX4 activity and lipid peroxidation treated in different groups, with or without US irradiation; Tumor volume/weight changes and survival rate after different treatments. Adapted with permission from 160, copyright 2021 PubMed Central; **(C)** Schematic illustration of structure, mechanism and treatment style of D-MOF(Ti); **(D)** The charge transfer of MOF(Ti) and D-MOF(Ti) enhances the mechanism diagram and corresponding energy band diagram of SDT; **(E)** Determination of ROS by EPR spectrum (up: ·OH , down: ^1^O_2_). Adapted with permission from 161, copyright © 2021 WILEY-VCH.

MOFs have attracted considerable attention in SDT due to their exceptional sonodynamic properties. Inspired by the development of MOFs for PDT, early SDT-oriented MOFs primarily focused on their intrinsic SDT properties rather than merely passive carriers for free sonosensitizers, while simultaneously enabling efficient drug loading of additional chemotherapeutics. For example, a manganese porphyrin-based MOF (Mn-MOF) was engineered to facilitate synergistic SDT and ferroptosis-mediated cancer therapy 160. In this system, Zr⁴⁺ ions served as coordination nodes and were bridged by manganese 5,10,15,20-tetrakis(4-benzoic acid) porphyrin (Mn-TCPP), which functioned as sonosensitizers. Notably, Mn^2+^ catalyzed the decomposition of H_2_O_2_ to generate O_2_, while Zr^4+^ depleted GSH, collectively promoting ROS accumulation and enhancing SDT efficacy (Figure 15A). Furthermore, US irradiation suppressed GPX4 activity and exacerbated ROS mediated ferroptosis (Figure 15B). Mn-MOF also exhibited potent anticancer effects by increasing the infiltration of activated CD8+ T cells and mature dendritic cells (DCs) while reducing myeloid-derived suppressor cells (MDSCs) within TME, thereby enabling a combined SDT-immunotherapy. TiO_2_ is a classical inorganic sonosensitizer; however, its ROS generation is severely limited by rapid electron-hole (e^-^/h^+^) recombination (50 ± 30 ns). In contrast, MOFs exploiting linker-to-metal-cluster charge transfer can significantly enhance charge transport and catalytic activity. A defective NH_2_-MIL-125 (Ti) (D-Ti-MOF), composed of Ti^4+^ clusters and 2-aminoterephthalic acid linker, was synthesized as one of the earliest highly effective a photocatalytic MOF 161. Subsequent hydrogen reduction generated Ti^3+^ (Figure 15C), resulting in bandgap narrowing, improved charge transfer, and enhanced e^-^/h^+^ separation, thereby markedly increasing SDT efficiency (Figure 15D). Moreover, the unique valence band (VB) and conduction band (CB) configuration enabled generation of ^1^O_2_ and hydroxyl radicals (•OH) via water catalysis, as confirmed by EPR spectra (Figure 15E).

In conventional sonosensitizers, metal-nitrogen moieties often serve as key active sites for sonoocatalysis. Inspired by this, a novel MOF featuring atomically dispersed transition-metal-nitrogen-coordinated Zn-Nx sites within the microporous carbon framework derived from ZIF-8 was developed 162. The unsaturated Zn-N active sites exposed on the (110) crystal facet enhanced electron transfer under US irradiation, effectively mimicking the functions of conventional sonosensitizers. These studies highlight that a critical future direction for SDT lies in the rational engineering of MOF structures to develop novel sonosensitizers with superior catalytic activity and therapeutic performance.

### MOFs for CDT based theranostics

CDT is an emerging anticancer modality that induces tumor cell apoptosis and necrosis by catalytically converting endogenous H_2_O_2_ into highly reactive •OH via the Fenton or Fenton-like reaction mediated by metal-based nanomaterials (*e.g.*, Fe, Cu, Mn, Cr). Compared with normal tissues, the TME exhibits is characterized by aberrant metabolism, resulting in a mildly acidic pH and elevated levels of H_2_O_2_—conditions that are particularly favorable for CDT 163. As a therapeutic strategy driven by endogenous stimuli, CDT exhibits high tumor selectivity and rational activation, highlighting its strong potential for clinical translation. Owing to their tunable architectures and intrinsic catalytic metal centers, MOFs are especially well suited for CDT applications.

**Figure 16:**
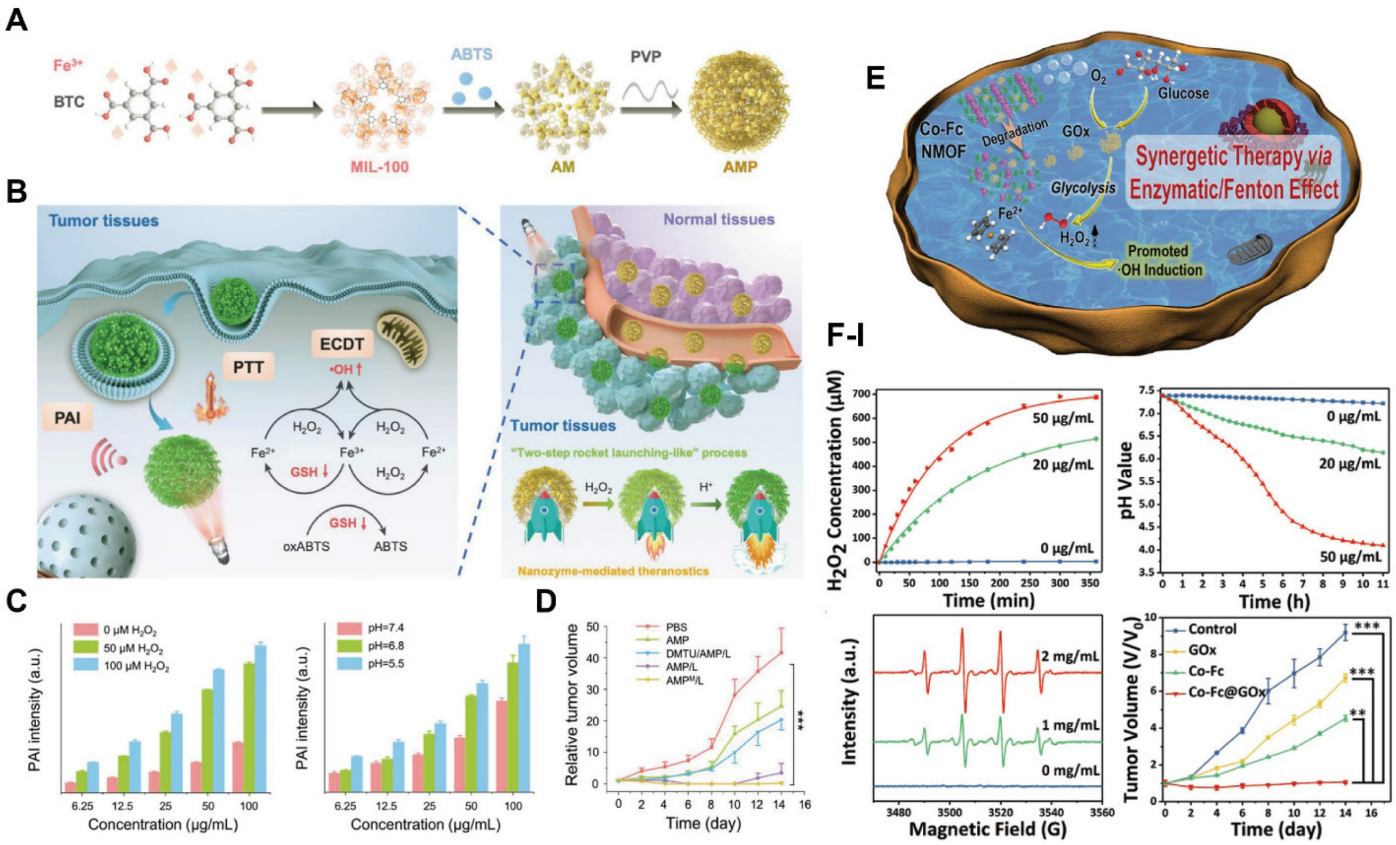
**(A)** Schematic diagram of AMP NPs preparation; **(B)** AMP as a dual-mode image-guided diagnostic reagent combination mechanism diagram (PTT-CDT); **(C)** PAI signal intensity under varying concentrations of H_2_O_2_ or at different pH levels; **(D)** The tumor volume changes. Adapted with permission from 164, copyright 2019 WILEY-VCH; **(E)** Schematic diagram of Co-Fc@GOx in synergetic therapy; **(F)** Changes in pH value of Co-Fc@GOx under different concentrations of glucose solution; **(G)** Changes in H_2_O_2_ concentration of Co-Fc@GOx under different concentrations of glucose solution; (H) EPR spectroscopy was used to detect the ability of Co-Fc@GOx to produce ·OH at different concentrations of glucose solutions; **(I)** The tumor volume changes in mice after treatments. Adapted with permission from 166, copyright 2020 WILEY-VCH.

For example, a MIL-100-based MOF loaded with 2,2'-azino-bis(3-ethylbenzothiazoline-6-sulfonic acid) (ABTS) and surface-modified with polyvinylpyrrolidone (PVP) was developed as a diagnostic and therapeutic nanoplatform for PA imaging-guided PTT and CDT combinational therapy (Figure 16A, B) 164. This system not only enabled efficient PAI but also promoted H_2_O_2_ activation under acidic conditions, amplifying the PAI signal (Figure 16C). Notably, the PAI intensity was detectable even in small tumors (~20 mm^3^), highlighting its promise for early tumor diagnosis. *In vivo* studies further validated the synergistic antitumor efficacy of the combined CDT-PTT therapy (Figure 16D).

Enhancing CDT efficacy generally relies on increasing endogenous H_2_O_2_ concentrations and further acidifying the TME. For instance, MIL-101(Fe)-based MOF incorporating juglone, a redox-active compound, significantly improved CDT performance by elevating intracellular H_2_O_2_ levels through an electron-transfer cascade, thereby enhancing Fenton reaction efficiency and tumor eradication 165. Similarly, a cobalt-iron bimetallic MOF (Co-Fe-MOF) with strong Fenton catalytic activity was developed and further functionalized with glucose oxidase (GOx) to construct a cascade nanoplatform (Figure 16E) 166. GOx catalyzes glucose oxidation within the TME, generating H_2_O_2_ and gluconic acid, which simultaneously increases H_2_O_2_ concentration and lowers pH. This synergistic coupling of enzymatic and Fenton reactions markedly enhanced CDT efficacy, as demonstrated by comprehensive *in vitro* and *in vivo* experiments (Figure 16F-I).

### MOFs for immunotherapy based theranostics

Cancer immunotherapy harnesses the immune system to selectively recognize and eradicate malignant cells, thereby inducing durable antitumor immunity and preventing metastasis and tumor recurrence. Currently, the major immunotherapeutic strategies include adoptive cell therapy, cancer vaccines, and immune checkpoint blockade therapy. Representative examples of adoptive cell therapy encompass chimeric antigen receptor (CAR) T-cell therapy, T-cell receptor-engineered (TCR) therapy and chimeric antigen receptor-natural killer cell (CAR-NK) therapy. Immune checkpoint blockade therapy functions by inhibiting immunosuppressive checkpoints, whereas cancer vaccines aim to elicit an immune response against tumor-associated antigens (TAAs) 167. In recent years, MOFs have attracted considerable attention as platforms for enhancing cancer immunotherapy, particularly when combined with external stimuli such as light, ultrasound, X-rays, or magnetic fields. Beyond direct tumor ablation, these externally activated MOF systems can induce immunogenic cell death (ICD), leading to the release of damage-associated molecular patterns (DAMPs) and TAAs. This process promotes DC maturation and T-cell activation, ultimately eliciting systemic antitumor immune responses. For example, an intracellular acid-responsive polymer-MOF hybrid (DIMP) was designed to co-deliver the chemotherapeutic agent DOX and the photo-therapeutic agent ICG for breast cancer therapy 168. This nanoplatform enabled precise and targeted drug delivery while effectively inducing ICD, enhancing DC maturation, and improving therapeutic efficacy, thereby achieving synergistic therapy. Such strategies also mitigated systemic toxicity and adverse effects associated with conventional pharmacological agents.

In addition to ROS-induced ICD, the direct incorporation of immune adjuvants into MOFs represents another promising approach for combination immunotherapy. For instance, a manganese-based MOF (Mn-MOF) electrostatically bound CpG oligonucleotides, which are Toll-like receptor 9 (TLR9) agonists functioning as potent immune adjuvants 169. Subsequently, a cancer cell membrane derived from B16 melanoma cells expressing the OVA antigen was coated on this complex to form cMn-MOF@CM. This biomimetic construct prolonged systemic circulation and enhanced tumor targeting. The synergistic effects of CpG-mediated immune stimulation and efficient SDT promoted ICD, DC maturation, and T-cell activation, ultimately generating robust antitumor immunity.

**Figure 17:**
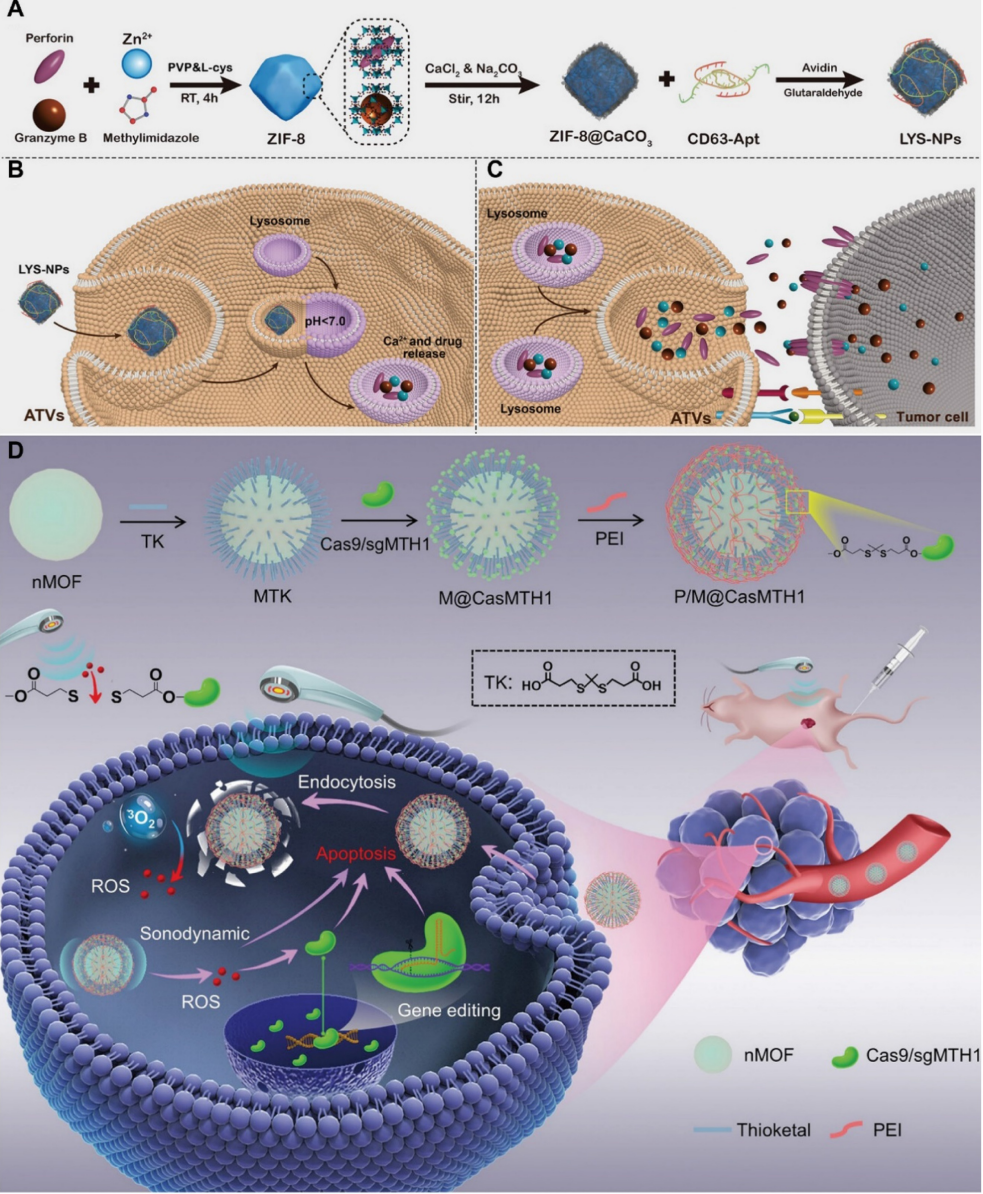
** (A)** Schematic illustration of LYS-NPs; **(B)** Lysosomal targeting and degradation of ATVs via CD63^+^ aptamer recognition; **(C)** ATVs releasing cytotoxic proteins and calcium ions in reconstructed lysosomes for tumor destruction. Adapted with permission from 170, copyright 2021 WILEY-VCH; **(D)** Schematic illustration of P/M@CasMTH1 for tumor treatment. Adapted with permission from 172, copyright 2021 WILEY-VCH.

In addition, MOFs have demonstrated considerable potential in facilitating adoptive T-cell therapies. A lysosome-responsive MOF nanoplatform (LYS-NP) was developed, which functioned as a TCR-based therapeutic strategy 170. These MOFs selectively targeted lysosomes within adoptive T cells, which subsequently functioned as intracellular carriers—termed adoptive T-cell vectors (ATVs)—for the controlled delivery of cytotoxic proteins. During fabrication, ZIF-8 frameworks were loaded with perforin and granzyme B, both of which are capable of inducing apoptosis in tumor cells. The MOF surface was further mineralized with calcium carbonate (CaCO_3_) and functionalized with a CD63 aptamer (CD63-Apt), enhancing biocompatibility and lysosomal targeting (Figure 17A). When the TCR was inactive, ATVs were retained within lysosomes in a quiescent state. Upon major histocompatibility complex (MHC)-mediated activation, lysosomal exocytosis was triggered, resulting in the localized release of perforin, granzyme B, and Ca²⁺ at the immunological synapse and effectively initiating localized tumor cell killing (Figure 17B, C). The CRISPR-Cas9 system is a versatile gene-editing platform that enables precise genomic modification, including knockout, knock-in, inhibition, and activation for cancer therapy. Stimuli-responsive and controlled release of the CRISPR-Cas9 complex is highly desirable to enhance therapeutic precision while minimizing genotoxicity 171. In this context, nanoscale MOFs have been utilized as efficient sonosensitizers to amplify SDT efficacy while simultaneously enabling ultrasound-triggered release of CRISPR-Cas9 ribonucleoproteins (RNPs) 172. Specifically, the CasMTH1 RNP complex was covalently conjugated to a porphyrin-integrated nMOF via ROS-responsive thioether linkages and subsequently coated with polyethyleneimine (PEI) to facilitate lysosomal escape (Figure 17D). Upon ultrasound irradiation, ROS generation cleaved the thioether bonds, triggering RNP release and disruption of the MutT Homolog 1 (MTH1) gene, a key enzyme responsible for detoxifying oxidized nucleotides and protecting tumor cells from oxidative stress. The synergistic combination of SDT-induced ROS production and CRISPR-mediated MTH1 gene disruption effectively undermined tumor cell defenses, leading to substantial inhibition of tumor progression both *in vitro* and *in vivo*.

### MOFs for other therapy-based theranostics

**Figure 18:**
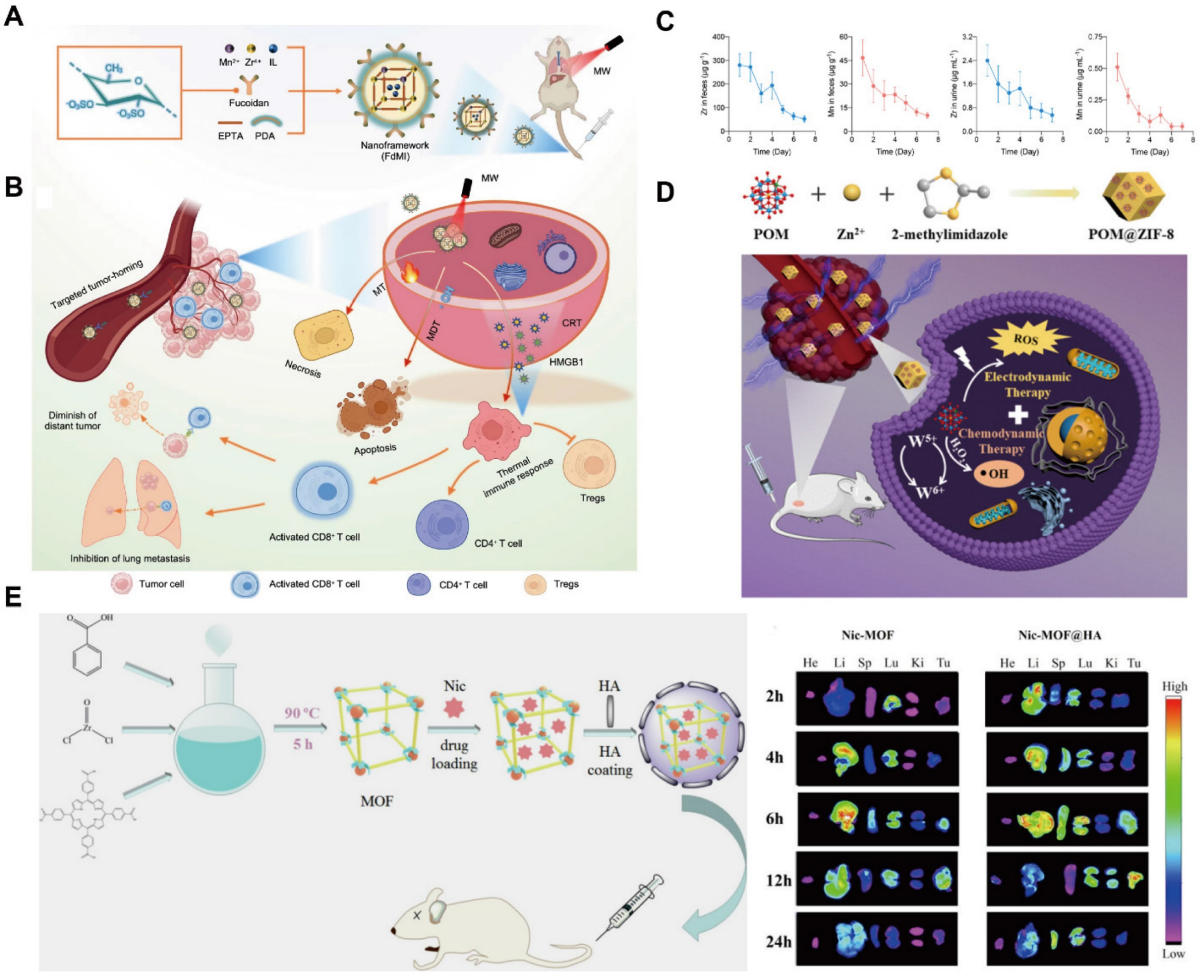
** (A)** Schematic synthesis of FdMI nanoframework; **(B)** Schematic diagram of the synergistic therapeutic mechanism of FdMI nanoframework; **(C)** Zr/Mn concentrations in feces/urine after *i.v.* injection of FdMI. Adapted with permission from 173, copyright 2024 American Chemical Society; **(D)** Schematic illustration of POM@ZIF-8 for EDT/CDT. Adapted with permission from 175, copyright 2022 American Chemical Society; **(E)** Synthesis of Nic-MOF@HA NPs and their biodistribution: representative ex vivo fluorescence images of major organs at different time points post-injection. (Tu: Tumor; He: Heart; Li: Liver; Sp: Spleen; Lu: Lung; Ki: Kidney). Adapted with permission from 176, copyright 2022 Elsevier.

In addition to the mainstream therapeutic strategies discussed above, MOFs have also been explored in several emerging cancer treatment modalities. Microwave thermotherapy (MT), a clinically established localized tumor ablation technique, induces tumor cell necrosis by exploiting the heat generated from the rapid rotation and friction of dipolar molecules under microwave irradiation 173. Compared with conventional surgical resection, MT offers several advantages, including reduced invasiveness, lower cost, and real-time intraoperative monitoring enabled by imaging guidance. To further improve the precision and efficacy of MT, tumor-targeted MOFs have been developed as microwave sensitizers capable of amplifying localized thermal effects within tumor sites. For instance, an Mn- and ionic liquid-doped MOF, following surface modification, was engineered as a highly efficient microwave sensitizer that effectively eradicated tumor cells via combined MT and microwave-dynamic therapy (MDT). Notably, microwave irradiation was found to stimulate antitumor immune responses, thereby enabling multimodal therapeutic synergy 173. Given the intrinsic metal content of MOFs, their *in vivo* metabolism and clearance were systematically evaluated to ensure biosafety (Figure 18A, B). Quantitative analysis of Zr and Mn in feces and urine within seven days after intravenous administration revealed a rapid decline over time, indicating efficient hepatic, biliary, and renal clearance, underscoring the favorable biocompatibility and safety profile of these MOFs (Figure 18C).

Electrodynamic therapy (EDT) represents another emerging modality in which electrical stimulation catalyzes the in-situ generation of ROS, enabling uniform tumor ablation across the entire electric field rather than being confined to regions near the electrodes. This approach is characterized by reliable therapeutic efficacy and high reproducibility 174. Beyond conventional electrocatalytic materials such as Fe_3_O_4_ and Pt, MOFs have attracted increasing attention as versatile nanoplatforms for EDT due to their structural tunability and multifunctionality. Moreover, the combination of EDT with CDT has demonstrated synergistic enhancement of ROS-mediated tumor ablation through complementary internal and external stimuli. Leveraging the pH-responsive properties of polyoxometalate-modified ZIF-8 (POM@ZIF-8) 175, POM can be released from the MOF matrix under acidic TME to function as an electrosensitizer. Within the applied electric field, POM generated ROS while simultaneously participating in Fenton-like reactions, thereby achieving potent and synergistic tumor eradication (Figure 18D).

Active targeting with MOFs is commonly achieved by functionalizing the surface of drug-loaded MOF nanoparticles with targeting ligands (such as antibodies, peptides, nucleic acid aptamers, polysaccharides, or hyaluronic acid) through physical or chemical means. This approach significantly enhances the precision of drug delivery to tumor cells, thereby improving therapeutic efficacy while minimizing off-target toxicity. For example, nicorandil (Nic) was encapsulated within MOFs, and HA was subsequently adsorbed on the MOF surface via electrostatic interactions (Figure 18E). Owing to the specific recognition of HA by the CD44 receptor overexpressed on tumor cells, Nic-MOF@HA exhibited significantly enhanced tumor accumulation throughout the observation period with non-HA-modified MOFs, confirming the effectiveness of active targeting and therapy 176. In addition to surface functionalization, the intrinsic properties of MOFs could also be exploited to achieve targeted treatment. In particular, MOFs enable stimulus-responsive drug release in response to tumor microenvironment-associated cues, allowing precise spatiotemporal control over drug delivery. Moreover, certain therapeutic agents exhibit activity only upon exposure to specific physical or chemical stimuli, for which MOFs serve as ideal carriers. This paradigm, referred to as stimulus-responsive targeting, including pH-responsive zinc- and iron-based MOFs, as well as ATP-responsive and GSH-responsive MOF-based drug delivery systems. These nanoplatforms primarily relied on endogenous TME responses, exogenous stimuli, such as magnetic field-responsive and thermosensitive MOFs have also been employed for targeted cancer therapy 177.

Enzyme therapy represents another promising non-invasive anticancer strategy, enabling the localized generation of cytotoxic species from non-toxic substrates without damaging normal tissues, thereby reducing the adverse effects associated with conventional therapies. For instance, enzyme-like catalytic activity can initiate cascade reactions that generates ROS, ultimately inducing tumor cell death 178. Manganese, an essential trace element, serves as a key cofactor in numerous metalloenzymes and nanozymes, including manganese catalase (MnCAT), manganese superoxide dismutase (MnSOD), and peroxidase (POD), and plays a critical role in inducing Fenton-like reactions. Recently, a Mn-based single-atom catalyst (Mn-N/C) was constructed by encapsulating coordinatively unsaturated monatomic Mn nanocatalysts within a ZIF-8 179. Benefiting from the large specific surface area and tunable pore structure of ZIF-8, the Mn-N/C catalyst exhibits high enzymatic activity for ROS generation while simultaneously activating the STING signaling pathway, thereby achieving potent antitumor therapeutic efficacy.

## Conclusion and perspectives

This review summarizes recent advances in MOF-based anticancer agents, highlighting their expanding applications across a broad spectrum of therapeutic modalities, including PDT, PTT, SDT, CDT, RT, gene therapy, immunotherapy, as well as multi-modal therapies over the past decade 180-183. These developments are largely driven by the remarkable structural tunability and functional versatility of MOFs. Substantial progress has been achieved in exploiting MOFs as DDS, molecular imaging agents, and multifunctional therapeutic platforms. The tunable synthetic parameters and unique physicochemical properties enable efficient encapsulation of diverse therapeutic agents, thereby advancing their utility as drug carriers, imaging probes, and integrated nanotheranostic systems. Notably, the integration of therapeutic and diagnostic functionalities within a single MOF-based platform holds promise for further enhancing precision nanomedicine and therapeutic efficacy 184-186.

Despite the encouraging advances, significant challenges remain on the path toward clinical translation. MOFs designed for biomedical applications must prioritize low toxicity and controlled degradability. While nanoscale dimensions (typically < 200 nm) improve circulation and tumor accumulation, they may also increase nanotoxicity risks, including inflammation and cell death. Importantly, MOF toxicity is highly composition-dependent. Ca-, Bi-, or Eu-MOFs generally exhibit minimal toxicity, whereas Ti-, Fe-, Co-, Al-, or Cr-MOFs display low to moderate, and Zr-, Mg-, Gd-, Ni-, Zn-MOFs demonstrate moderate toxicity. Among reported systems, Fe-MOFs are the most extensively studied, reflecting their biomedical relevance. Toxicity mechanisms include reactive species generation by redox-active metals (*e.g.*, Ag, Cu, Fe, Cd), ion-homeostasis disruption (*e.g.*, Ga, Fe, Zn, Cd), and insoluble-protein accumulation (*e.g.*, Pb, Mn, Ag, Co). Organic linker toxicity is primarily associated with membrane permeability, with carboxylate-based linkers generally exhibiting lower toxicity than azolate ligands. Surface charge further influences toxicity, as positively charged surfaces enhance cellular uptake but also increase cytotoxic risk 187. Additionally, residual solvents and additives from MOF synthesis may pose safety concerns, underscoring the need for green synthesis.

Degradability in biological environments is another critical design factor for biomedical MOFs. While nanosizing enhances therapeutic performance, it often compromises structural stability, as increased surface area accelerates interactions with biological media and promotes degradation. MOF stability could be partially predicted using the Hard and Soft Acids and Bases (HSAB) principle: stable MOFs generally comprise carboxylate ligands (hard Lewis bases) coordinated with high-valent metal ions (hard Lewis acids). Alternatively, strong coordination can also arise from azolate-based ligands paired with low-valent transition metals. Additional factors influencing stability include metal cluster nuclearity, ligand coordination strength, chelating group number and type, defect density, open metal sites, and the hydrophilic-hydrophobic balance of the framework.

Hydrophilic surfaces generally reduce aggregation and improve dispersion, whereas hydrophobic MOFs exhibit stronger serum protein adsorption, leading to accelerated clearance and reduced stability. Moreover, MOF physicochemical properties, particularly surface charge and chemistry, further govern colloidal and chemical stability in biological fluids. A careful balance between toxicity and degradability is therefore essential. Rapid degradation may increase the release of metal ions and organic ligands, elevating toxicity risks. Consequently, MOF nanoparticles should ideally be constructed from low-toxicity components. MOF degradation does not always yield the original building blocks; instead, materials may undergo recrystallization, amorphization, or transformation into metal oxides or insoluble phosphates, which may not induce immediate toxicity 188. Appropriate dosing must be rigorously optimized, and long-term safety validated through extended *in vivo* studies. Establishing robust quality control standards is also critical to ensure batch-to-batch reproducibility and scalability. Moreover, key translational challenges-such as controlled release in clinical settings, metal ion excretion pathways, and long-term biosafety-require systematic investigation. Encouragingly, several MOF-based systems, including ZIF-8 and PCN-222, have advanced to preclinical studies 189. However, concerns remain regarding the stability of ZIF-8 under physiological pH, particularly the risk of premature drug leakage and potential neurotoxicity resulting from sustained zinc ion release. Notably, the MOF-based radiosensitizers RiMO-301 (NCT0583-8729, NCT03444714) and RiMO-401 (NCT06182579) have progressed to Phase II and Phase I clinical trials, respectively, for the treatment of head and neck cancer and advanced solid tumors 190. Their well-defined mechanisms of action and small-molecule-based manufacturing processes facilitate translational development. In particular, the clinical progress of RiMO-301 is expected to significantly influence confidence and investment across the broader MOF research landscape.

Overall, the requirements for MOFs in biomedical applications are substantially more stringent than those for industrial uses. Prior to clinical implementation, systematic and advanced investigations are essential to elucidate the relationships among their toxicity, biodistribution, and intrinsic material properties, including composition, particle size, stability, morphology, surface chemistry, and administration routes. Integrating comprehensive *in vitro* and *in vivo* evaluations with advanced modeling approaches will enable a more holistic understanding of MOF-biological interactions.

At present, the clinical translation of MOF-based therapeutics remains at an early stage, with several unresolved challenges: 1. Complexity of multifunctional design and potential toxicity. While multifunctional MOF nanoplatforms enable targeted imaging and therapy, their structural complexity and reliance on heavy metals hinder scalability and raise safety concerns. Simplified designs and scalable synthesis strategies are therefore required to bridge laboratory research and clinical application 191-193. Surface modification with ligands, antibodies, or peptides improves targeting specificity but remains difficult to reproduce with consistent biocompatibility 194,195. 2. Biosafety and biocompatibility concerns. Most existing studies focus on short-term toxicity 196, whereas long-term toxicity, immune compatibility, and bioaccumulation remain insufficiently explored. Strategies incorporating endogenous or low-toxicity metal ions and biomolecule-derived ligands may improve safety profiles. 3. Biological metabolism and clearance. Comprehensive understanding of MOF degradation, metabolism, and excretion pathways is critical to prevent chronic accumulation and adverse outcomes 197.

Given these challenges, future research should prioritize the development of simpler, environmentally sustainable, and multifunctional MOF platforms that maintain high porosity and tunable architecture while enhancing stability, circulation time, and biosafety. Understanding how degradation behavior influences drug release, tumor accumulation, and systemic tolerance will be central to rational MOF design. In addition, the subchronic and chronic toxicity of degradation products-both metal ions and organic linkers-requires detailed molecular-level investigation. To accelerate clinical progress, several strategic priorities should be addressed. First, the development of advanced *in vivo* tools to monitor MOF biodistribution and pharmacokinetics, along with standardized frameworks for evaluating absorption, distribution, metabolism, and excretion (ADME), is needed. Additionally, rigorous validation of safety and efficacy to bridge preclinical and clinical studies 198. Finally, the application of design principles derived from clinically approved nanomedicines.

In conclusion, MOFs represent a transformative platform for next-generation antitumor therapeutics. Their unique ability to integrate drug delivery, imaging, and therapeutic functions within a single modular system positions them as powerful candidates for precision oncology. Although substantial translational challenges remain, the rapid progress achieved to date highlights the immense potential of MOF-based nanoplatforms for future cancer diagnosis and therapy.

## Figures and Tables

**Table 1:** Typical synthetic approaches of MOFs

Synthesis Methods	Energy source	MOFs	Approx. Reaction time	Approx. Temperature	Advantage	Disadvantage	Ref.
Solvo/Hydrothermal	Thermal; Electric source	MIL-88A-Fe; MOF-5-Zn; ZIF-8-Zn	4-96 h	50-1000 °C	High yields; Good crystallinity; High porosity	Time-consuming; Toxic	32-34
Microwave-heated	Electromagnetic wave	MIL-100-Fe; MIL-101-Cr	5 min-4 h	30-150 °C	Shortening of reaction time; Narrow particle size distribution; High synthesis rate; Controllable particle size	Low productivity; High costs; Small synthesis scale	35,36
Sonochemical	Ultrasound	[Gd_2_(TATAB)_2_]·6DMF; MIL-88A (Fe)	5 min-2 h	25-50 °C	Small size; Green and efficient energy	Restricted temperature range	37,38
Electrochemical	Direct current power	HKUST-1 (Cu) ; [Zn(1,3-bdc)0.5(bzim)]	10 min-1 h	Room Temperature	Mild; Short reaction time; Continuous synthesis of controllable morphology	Requires special equipment	39,40
Mechanochemical	Mechanical force	HKUST-1-Cu; MOF-14-Cu	0.5 h-6 h	Room Temperature	Green; Low costs; High yields	Limited to specific MOFs; Might lead to the poor crystallinity	41,42
Reverse-Phase Microemulsions	-	DBP-UiO (Hf); Mn_3_(BTC)_2_(H_2_O)_6_	0.5 h-24 h	Room Temperature - 80 °C	Pure; Synthesis of controllable	Post-processing complex; High costs	43,44

**Table 2:** Summary of MOFs-based drug delivery systems for chemotherapy.

MOFs	Drug	Loading percentage (wt%)	Release	Ref.
MIL-53-Cr	ibuprofen	22.0	18 days (SBF)	92
MIL-53-Fe	ibuprofen	21.0	20 days (SBF)	
	caffeine	29.2	6 h (SBF)	93
	busulfan	18.0	8 h (phosphate-buffered saline, PBS)	94
MIL-100-Cr/MIL-101-Cr	ibuprofen	25.8/58.0	3/6 days (SBF)	96
AuNR@ZIF-8-Zn	DOX	26.3	12 h (PBS, 95%)	100
AuNR@ZIF-8-Zn Janus	DOX	30.0	24 h (PBS, > 80%)	101
CAD@ZIF-8-FA (Zn)	DOX	34.75	96 h (PBS)	102
ZIF-8-Zn	5-FU	45.4	7 days (PBS)	98
	DOX	4.7	30 days (66%, DI Water)	99
	rapamycin	9.4	96 h (about 86%, PBS)	103
	camptothecin	26.3	15 h (76.3%, PBS)	104
	caffeine	28.0	27 days (SBF)	105
Fe-Zn-ZIF-8	5-FU	15.7	24 h (about 76%, PBS)	106
NiCo-Tb-PEGMA-AS1411	DOX	60.3	48 h (52.8%, PBS)	107

**Table 3:** Summary of MOFs-based gene drug delivery systems (cargo, loading rate, MOFs materials, MOFs diameter, and application).

Cargo	MOFs	Materials	Loading rate (wt%)	Diameter (nm)	Application	Ref.
DNA	DNA@UiO-66-N3	UiO-66 (Zr)	12.3/13.0 pmol cm^-2^	14/19	synthesis	109
DOX-SOR-DNA	ZIF-67@DS@ext-DNA	ZIF-67 (Co)	59.7/60.2	100-1000	therapy for MCF-7 cells	114
HP-SS-BT (DNA)	HP-SS-BT@UiO66-NH_2_	UiO-66 (Zr)	72.3	115	nanoprobe	115
pDNA	pEGFP-C1@ZIF-8	ZIF-8 (Zn)	3.4	275.7	therapy for MCF-7 cells	110
siRNA	ICG@ZIF-8@siRNA	ZIF-8 (Zn)	8.15	166	therapy for A549 cells	111
siRNA	P-MOF-siRNA(Zn)	ZIF-8 (Zn)	/	175	therapy for SK-BR-3 cells	112
miRNA	miR-34a-m@ZIF-8	ZIF-8 (Zn)	3.6	255	therapy for MDA-MB-231 cells	113
mRNA	MOF-PGMA(EA)	UiO-66 (Zr)	/	26.4/41.7	drug delivery	116

**Table 4:** **The applications of MOFs for molecular imaging** (imaging modality, MOFs, cargo, cell line, and application).

Imaging Modality	MOFs	Cargos	Cell line	Applications	Ref.
FL/MRI/PAI	MIL-100-Fe	ICG (40%)	MCF-7	Imaging (r_2_: 54 mM^-1^s^-1^), PDT	119
FL/MRI	Fe-TCPP	DHA (75%)	4T1	Imaging, PDT, Chemotherapy	120
MRI	Gd/MPC	aPD-1 (28.23%)	SCC7	Imaging, Immunotherapy (Tumor inhibition rate: 84.6%)	122
MRI/FL	UiO-66(Zr)-(COOH)_2_	DOX	4T1	Imaging (r_1_: 18.77 mM^-1^s^-1^), Chemotherapy	123
MRI/FL	Mn-TCPP	-	4T1	Imaging (r_1_: 6.08 mM^-1^s^-1^), PDT	124
MRI	Zr-MOF@MnO_2_@Tm	Apatinib (32.9%)	4T1	Imaging, PDT, Chemotherapy	125
MRI	MnO_x_/UiO-66-F/PPEG	-	4T1	Imaging (r_1_: 11.16 mM^-1^s^-1^)	126
MRI	Fe_3_O_4_@UiO-66-Zr@WP6	5-FU (0.83 µmol mg^-1^)	Hela	Imaging (r_2_: 72.23 mM^-1^s^-1^), Chemotherapy	127
IP/MRI	PPy@MIL-53-Fe/DOX	DOX (90%)	4T1	Imaging, PTT, Chemotherapy	128
MRI/FL	Mn_3_[Co(CN)_6_]_2_@MIL-100@AS	Artesunate (53%)	Hela	Imaging (r_1_: 6.61 mM^-1^s^-1^, r_2_: 76.24 mM^-1^s^-1^), Chemotherapy (Tumor inhibition rate: 82.8%)	129
MRI	UCNP@Fe-MIL-101_NH_2_	-	KB	Imaging (r_2_: 67.32 mM^-1^s^-1^)	130
CT	Cu(I_4_-BDC)/Zn(I_4_-BDC)	-	-	-	131
CT	UiO(Zr)-PDT	BODIPYs	Walker-256	Imaging (CT value: 92 HU), PDT	132
CT	DOX@LA-AuNR/ZIF-8	DOX (30%)	H-22	Imaging, PTT, Chemotherapy (Tumor inhibition rate: 93%)	101
CT/MRI/PA	GNR-MSNs-MA-Fe	-	4T1	Imaging, PTT, Chemotherapy	133
PET	DOX@^64^Cu-MOF-Au-PEG	DOX	U87MG	Imaging, RT, Chemotherapy (Tumor inhibition rate: 89%)	135
PET/FL	DOX@^89^Zr-UiO-66/Py-PGA-PEG-F3	DOX (50%)	MDA-MB-231	Imaging, Chemotherapy	136
PET	Zr-MOF-PAC	DOX (25.1 µmol g^-1^)	U87MG	Imaging	137
PAI	Au@ZIF-8	-	4T1	Imaging, PTT (Tumor apoptosis: 77.48%)	139
PAI/MRI/CT	Au@MIL-88(A)	-	U87MG	Imaging	140
PAI/IR	Au@MOF-DOX	DOX (29%)	H22	Imaging, PTT, Chemotherapy	141

**Table 5:** ** The application of MOFs for theranostics** (cargo, modifier, imaging, properties, and applications).

MOFs	Cargo (wt %)	Modifier	Imaging	Properties and Applications	Ref.
Hf-DBP/Bi-DBP	-	-	-	RT/RDT for TRAMP-C2/Panc02 tumor	146
Hf-HCBB/MSA-2	MSA-2 (29.2%)	-	-	RT for CT26 tumor	147
Oxy-HB@HP (Hf)	Oxygen	Hemoglobin(48.9%)	-	RT/RDT for CT26 tumor	148
ICG@ZIF-8	ICG (10.2%)	-	FL	PTT for SMMC-7721 tumor	149
pDA/MTX@ZIF-8	MTX (16.5%)	pDA	-	IC50: 8.27 µg mL^-1^, PTT/chemotherapy for MG63 tumor	150
HMPB (Fe)@PEI/ICG/DOX	ICG/DOX (32.12%/40.46%)	PEI	FL/IR	Photothermal conversion efficiency: 45.51%, Tumor inhibition rate: 95.5%, PTT/PDT/chemotherapy for 4T1 tumor	151
PS@MIL-100-Fe-F127	Ce6/TPEDC/TPETCF (42%/49%58%)	F127	-	PDT for 4T1 tumor	152
DBP-UiO (Hf)	-	-	-	PDT for SQ20B tumor	153
HA-DOX-PCN	DOX (51.9%)	HA	-	PDT for Hek-297T/SCC7/MDA-MB-231 cells	154
Fe-TCPP@BSA/SDs@MnO_2_	MnO_2_	BSA/SDs	MRI (r_1_: 6.09 mM^-1^s^-1^)	PDT for 4T1 tumor	155
CM-MMNPs (Zr)	MnO_2_ nanosheet	Cancer cell membrane	MRI	PDT for 3T3 tumor	156
MnFe_2_O_4_@MOF (Zr)	-	PVP	MRI	PDT for 4T1 tumor	157
MOFs (Fe)@SF@TPZ	TPZ (7.7%)	silk fibroin	FL	Tumor inhibition rate: 99.6%, PDT/CDT/chemotherapy for 4T1 tumor	158
Mn-MOF (Zr)	-	-	-	Tumor inhibition rate: 89.2%, pulmonary nodules inhibition rate: 96.9%, SDT/immunotherapy for H22 tumor	160
D-Ti-MOF	-	-	-	SDT/CDT for 4T1 tumor	161
Zn-N_3_-ZIF-8	-	-	-	Tumor inhibition rate: 84.6%, SDT for 4T1 tumor	162
ABTS@Fe-PVP	ABTS (22.0%)	PVP (25.3%)	PA/IP	CDT/PTT for 4T1 tumor	164
JMIL-101-Fe@CM	Juglone (10.2%)	Cell membrane (80.3±5.1 μg mg^-1^)	-	Tumor inhibition rate: 77.0%, CDT/chemotherapy for PC-3 tumor	165
Co-Fc (Fe)@GOx	GOx (1%)	PVP	-	CDT/starvation therapy for 4T1 tumor	166
ZIF-8 (Zn)@ICG@DOX@PVP	ICG/DOX (16.84%)	PVP	FL/PA/IP	PTT/PDT/chemotherapy/immunotherapy for 4T1 tumor	168
cMn-MOF@CM	CpG (20%)	B16-OVA membrane	-	SDT/immunotherapy for B16-OVA tumor	169
LYS-NPs (Zn)	Perforin/ Granzyme B (71.36%)	CD63- Aptamer	FL	Tumor inhibition rate: 70.9%, Immunotherapy for A549 tumor	170
P/M@CasMTH1 (Zr)	Cas9/sgMTH1	Thioglycolald-ehyde/PEI	FL	ROS level: 83.0%, Tumor inhibition rate: 60%, SDT/immunotherapy for A549 tumor	172
FdMI (Mn/Zr)	-	PDA/fucoidan	FL	Tumor inhibition rate: 78.7%, MT/MDT for 4T1 tumor	173
POM@ZIF-8 (Zn)	-	POM	-	EDT for Hela tumor	175
Nic-MOF@HA (Zr)	Nic (24.12%)	HA	FL	Tumor inhibition rate: 95.66%, Target therapy for 4T1 tumor	176

## Abbreviations

MOFs: Metal-Organic Frameworks
SBUs: Secondary Building Units
COFs: Covalent Organic Frameworks
IUPAC: International Union of Pure and Applied Chemistry
siRNA: short interfering RNA
FL: Fluorescence Imaging
MRI: Magnetic Resonance Imaging
CT: Computed Tomography
PET: Positron Emission Tomography
PAI: Photoacoustic Imaging
PS: Photosensitizers
PDT: Photodynamic Therapy
RT: Radiotherapy
TME: Tumor Microenvironment
EPR: Enhanced Permeability and Retention effects
LAG: Liquid-assisted Grinding
ILAG: Ion- and Liquid-assisted Grinding
SPCNs: Spherical Porous Carbon Nanoparticles
SACs: Single-atom Catalysts
AI: Artificial Intelligence
CSD: Cambridge Structural Database
LbL: Layer-by-layer
CTAB: Cety-ltrimethylammonium Bromide
FA: Folic Acid
HA: Hyaluronic Acid
PEG: Polyethylene Glycol
O_2_: Oxygen
PDA: Polydopamine
Ce6: Chlorin e6
DDS: drug delivery systems
DOX: Doxorubicin
AIDS: Acquired Immune Deficiency Syndrome
PBS: Phosphate-buffered Saline
IBU: Ibuprofen
SBF: Simulated Body Fluid
5-FU: 5-Fluorouracil
CAA: Cis-aconitic-anhydride
GSH: Glutathione
pDNA: Plasmid DNA
RNAi: RNA interfere
mRNA: Messenger RNA
miRNA: Micro RNA
ICG: Indocyanine Green
IP: Infrared-Photothermal
FDA: Food and Drug Administration
DHA: Dihydro-artemisinin
aPD-1: anti-Programmed Death-1
SPIONs: Superparamagnetic Iron Oxide Nanoparticles
Py: Pyrrole
PPy: Polypyrrole
UCL: Upconversion Luminescence
GNRs: Gold Nanorods
LA: Lactobionic Acid
NIR: Near-infrared
PTT: Photothermal Therapy
OVA: Ovalbumin
CpG ODNs: Cytosine-phosphate-guanine Oligodeoxy-nucleotides
TLR7/8: Toll-like Receptor 7/8
CDT: Chemodynamic Therapy
SDT: Sonodynamic Therapy
STING: Stimulator of Interferon Genes
RDT: Radiodynamic Therapy
^1^O_2_: Singlet Oxygen
Hb: hemoglobin
MTX: Methotrexate
ROS: Reactive Oxygen Species
H_2_O_2_: Hydrogen Peroxide
TPZ: Tirapazamine
HAP: Hypoxic-activated Precursor
DC: Dendritic Cell
MDSCs: Myeloid-derived Suppressor Cells
VB: Valence Band
CB: Conduction Band
•OH: Hydroxyl Radicals
ABTS: 2,2'-azino-bis(3-ethylbenzothiazoline-6-sulfonic acid)
PVP: Polyvinylpyrrolidone
GOx: Glucose Oxidase
CAR: Chimeric Antigen Receptor
TCR: T-cell Receptor-engineered
NK: natural killer
TAAs: Tumor-associated Antigens
ICD: Immunogenic Cell Death
DAMPs: Damage Associated Molecular Patterns
Mn-MOF: Manganese-based MOF
TLR9: Toll-like Receptor 9
ATVs: Adoptive T cell Vectors
CaCO_3_: calcium carbonate
MHC: Major Histocompatibility Complex
RNP: Ribonucleoprotein
PEI: Polyethyleneimine
MTH1: MutT Homolog 1
MT: Microwave Thermotherapy
MDT: Microwave-dynamic Therapy
EDT: Electrotherapy
POM: polyoxometalate
Nic: Nicorandil
MnCAT: Manganese Catalase
MnSOD: Manganese Superoxide Dismutase
POD: Peroxidase
HSAB: Hard and Soft Acids and Bases
